# The evolution of a series of behavioral traits is associated with autism-risk genes in cavefish

**DOI:** 10.1186/s12862-018-1199-9

**Published:** 2018-06-18

**Authors:** Masato Yoshizawa, Alexander Settle, Meredith C. Hermosura, Lillian J. Tuttle, Nicolas Cetraro, Courtney N. Passow, Suzanne E. McGaugh

**Affiliations:** 1 Department of Biology, University of Hawai‘i at Mānoa, 2538 McCarthy Mall, EDM 216, Honolulu, HI 96822 USA; 2 Department of Cell and Molecular Biology, School of Medicine, University of Hawai’i at Mānoa, Honolulu, HI 96813 USA; 3 Department of Ecology, Evolution, and Behavior, University of Minnesota, St. Paul, MN 55108 USA

**Keywords:** Vertebrate model, hapFLK, Systemic regulation, Psychiatric disease, Adaptation, *Astyanax mexicanus*

## Abstract

**Background:**

An essential question in evolutionary biology is whether shifts in a set of polygenic behaviors share a genetic basis across species. Such a behavioral shift is seen in the cave-dwelling Mexican tetra, *Astyanax mexicanus*. Relative to surface-dwelling conspecifics, cavefish do not school (asocial), are hyperactive and sleepless, adhere to a particular vibration stimulus (imbalanced attention), behave repetitively, and show elevated stress hormone levels. Interestingly, these traits largely overlap with the core symptoms of human autism spectrum disorder (ASD), raising the possibility that these behavioral traits are underpinned by a similar set of genes (i.e. a repeatedly used suite of genes).

**Result:**

Here, we explored whether modification of ASD-risk genes underlies cavefish evolution. Transcriptomic analyses revealed that > 58.5% of 3152 cavefish orthologs to ASD-risk genes are significantly up- or down-regulated in the same direction as genes in postmortem brains from ASD patients. Enrichment tests suggest that ASD-risk gene orthologs in *A. mexicanus* have experienced more positive selection than other genes across the genome. Notably, these positively selected cavefish ASD-risk genes are enriched for pathways involved in gut function, inflammatory diseases, and lipid/energy metabolism, similar to symptoms that frequently coexist in ASD patients. Lastly, ASD drugs mitigated cavefish’s ASD-like behaviors, implying shared aspects of neural processing.

**Conclusion:**

Overall, our study indicates that ASD-risk genes and associated pathways (especially digestive, immune and metabolic pathways) may be repeatedly used for shifts in polygenic behaviors across evolutionary time.

**Electronic supplementary material:**

The online version of this article (10.1186/s12862-018-1199-9) contains supplementary material, which is available to authorized users.

## Background

Animal species have evolved to changing environments by modifying morphological, physiological and behavioral outputs [1]. One challenging question in evolutionary biology is how animals evolved multiple behaviors with independent genetic bases. Indeed, some behavioral syndromes demonstrate that correlated behaviors can be underpinned by different genetic factors [2, 3]. Currently, it is largely unknown if any particular suite of genes, or so-called ‘genetic toolkit’ [4–6], are modified across evolutionary time to affect a set of genetically independent multiple behaviors.

To investigate how multiple behaviors evolve, we focused on behaviors whose physiological and molecular pathways may be comparable across species. For example, almost all animal species exhibit a sleep-like state, characterized by extended periods of behavioral quiescence that correlate with elevated arousal thresholds to sensory stimuli [7, 8]. Moreover, the molecular (e.g. melatonin) and cellular mechanisms of sleep-like states are shared among some animal species [9, 10]. Similarly, despite large differences in the complexities of their behavioral traits, vertebrates share many core characteristics of neural connectivity and molecular pathways in their innate social behaviors (e.g. mesolimbic system and oxytocin/vasopressin) [6, 11–15], stress-related behaviors [16], attention/cognition-related molecular pathways [17], and starvation/satiation pathways [18]. Some of these behaviors, including social responses, show even deeper conservation within Metazoa, especially in molecular pathways [5, 6]. This conserved molecular pathway was suggested as a genetic ‘toolkit’ for repeated evolution of social behavior [6].

Since many of these behavioral pathways are shared across vertebrates, we examined the Mexican teleost *Astyanax mexicanus,* which consists of both cave-dwelling and surface-dwelling populations. Notably, the cave morphs have significantly diverged from the surface morphs in multiple behaviors during evolution. Such traits include loss of schooling (reduced social interaction), performing repetitive behaviors, sleep deficits, hyperactivity, behavioral adherence to a particular vibration stimulus at 40 Hz (behaviorally adhere to a particular stimulus, called vibration attraction behavior, or VAB [19]), and higher cortisol levels (related to higher anxiety levels) [20]. Unlike cavefish, the conspecific surface-dwelling populations readily school, do not exhibit repetitive behavior or hyperactivity, have normative sleep, do not show strong adherence to a vibration stimulus, and have lower cortisol levels than cave morphs. This polymorphic suite of traits that are present in one ecotype and absent in another is rare within that natural world. Surprisingly, many of these cavefish behaviors overlap with the core symptoms of a human psychiatric disease, autism spectrum disorder (ASD) (e.g., reduced social interaction, performing repetitive behavior, sleep deficits, hyperactivity, adherence to a particular stimulus or object, and higher anxiety level [21–23]). In addition, many of these ASD-like traits in cavefish show large variations, ranging from the levels of surface- to cave-type [19, 24, 25]. This is reminiscent of the large variation within ASD: from severe to high functioning [22]. Accordingly, the behavioral similarities and the homologies of the vertebrate nervous system motivated us to investigate whether shifts in orthologs of ASD-risk genes may underpin the evolution of multiple behaviors of both humans and a teleost species.

This study, therefore, seeks to answer the following questions: (1) Are ASD risk genes in humans and *A. mexicanus* expressed in similar directions when comparing ASD patients with human controls, and cavefish with surface fish? (2) Do human ASD-risk genes exhibit signatures of molecular evolution in cavefish that are divergent from the rest of the genome and may indicate selection? (3) Do cavefish respond to pharmacological treatments for autism in a similar way as patients, suggesting a shared neural basis in the regulation of ASD-like behaviors (e.g. dopaminergic, serotonergic, adrenergic circuits) [26–29]?

## Results

We first queried the *Astyanax* genome to identify orthologs of the ASD-risk genes, which are listed in the database of Simons Foundation Autism Research Initiative (SFARI) (sfari.org) [30]. We found that 92.5% of 493 human ASD-risk genes (SFARI Gene database Category 1 to 4 and Category S—high evidenced ASD-risk genes—accessed in March 2017 [20, 30–32]. See Methods) have orthologs in the *Astyanax mexicanus* genome v1.2 (Table 1, Additional files 1 and 2).

**Table 1:** The enrichment of the expression shifts between surface fish and cavefish in ASD-risk genes

Human ASD-risk genes		Cavefish genes				
Risk category	# of Listed Genes in Sfari.org	% (#) human ASD-risk genes with cavefish orthologs	% (#) of orthogroups that show significant age × morph interaction†	% (#) of orthogroups that show significant expression difference between morphs at 72 hpf†	% (#) of all paralogs that show significant age × morph interaction†	% (#) of all paralogs that show significant expression difference between morphs at 72 hpf†
Category 1	19	94.7% (18 of 19)	72.2% (13 of 18)	94.4% (17 of 18)	61.1% (**> 99.9 percentile**) (22 of 36)	75.0% (**> 99.9 percentile**) (27 of 36)
Category 2	43	93.0% (40 of 43)	65.0% (26 of 40)	67.5% (27 of 40)	60.7% (**> 99.9 percentile**) (34 of 56)	64.3% (**> 99.9 percentile**) (36 of 56)
Category 3	139	95.0% (132 of 139)	60.6% (80 of 132)	70.5% (93 of 132)	50.3% (84.8 percentile) (95 of 189)	58.2% (**> 99.9 percentile**) (110 of 189)
Category 4	244	89.8% (219 of 244)	62.6% (137 of 219)	63.0% (138 of 219)	51.9% (96.1 percentile) (167 of 322)	52.5% (94.4 percentile) (169 of 322)
Category S (not already included among Cat 1–4 genes)	48	97.9% (47 of 48)	57.4% (27 of 47)	63.8% (30 of 47)	44.2% (4.7 percentile) (34 of 77)	49.4% (59.0 percentile) (38 of 77)
Total	493	92.5% (456 of 493 human genes)	62.1% (283 of 456)	66.9% (305 of 456)	53.8% (352 of 654)	54.6% (357 of 654)
Bootstrapping score: mean ± 95% confidence interval					48.0 ± 4.2%	49.0 ± 4.4%

The 72 h post-fertilization (hpf) represents the stage in which fish have hatched, but have not yet developed a swim bladder and the jaw is underdeveloped, comparable to the late embryonic stage of mammals [111]. † P < 0.05 after Benjamini-Hochberg adjustment. Percentiles in the tables are from 9999-bootstrapped values. SF: surface fish. CF: Pachón cavefish. Hpf: hours post fertilization. We avoided testing the differences between morphs in each developmental time point (i.e. 10, 24 and 36 hpf) due to save statistical power. The number of genes are indicated in parentheses. See also Additional file 1 for each statistical test of age x morph and expression difference at 72 hpf

In human studies, some ASD-risk genes exhibit differential expression between people with and without ASD [33–35]; thus, we analyzed gene expression differences between cavefish and surface fish using a previously published RNAseq dataset for *A. mexicanus* [36, 37]*.* This dataset includes gene expression data collected from whole individuals of both surface fish and Pachón cavefish at key developmental time points: 10 h post-fertilization (hpf; end of the gastrulation), 24 hpf (end of somitogenesis; hatching), 36 hpf (live with yolk) and 72 hpf (most of the organs, including gut and jaw, have developed) (GenBank SRA; accession code: PRJNA258661 [31, 36, 38]). Since ASD symptoms in humans emerge at an early developmental stage (even before 1–2 years old [32, 39]), we investigated both the interaction of age × morph (surface fish and cavefish) for all time points and the expression difference between morphs at 72 hpf in ASD-risk genes. We hypothesize that this 72 hpf is a comparable time point to ‘just before birth’ in humans when the basic neural circuit has been formed and is ready to prune synapses and rewire to form the proper neural circuits in response to environmental stimuli [40, 41].

Remarkably, genes in the categories with stronger evidence of association with ASD in humans (Categories 1 and 2 in SFARI Gene), were more often significantly differently expressed between surface fish and cavefish than in categories with weaker evidence of association with ASD in humans (Categories 3, 4 in SFARI Gene) (Table 1, Additional files 1 and 2). This trend was observed in both the interaction of age × morph (Table 1: Cat. 1–2 range 65–72% vs. Cat. 3-S range 57–63%) and the expression difference between morphs at 72 hpf (Table 1: Cat. 1–2 range 68–94% vs. Cat. 3-S range 63–71%) of orthogroups. Note, an orthogroup consists of multiple paralogs that share the same ancestor with each human gene [42]. This trend of higher rates of differential expression for genes in categories with stronger evidence was also seen at the level of individual *A. mexicanus* genes (i.e., paralogs; Table 1, Additional file 1). This suggests that, although paralogs evolved from gene duplication events and may be under different expression regulation, differentially expressed orthologs of ASD-risk genes are more common in the higher confidence SFARI genes.

To test whether the observed levels of differential expression for orthologs of Category 1 and 2 are significantly higher than for a random subset of genes across the *Astyanax* genome, we performed bootstrapping using 9999 random samplings of 500 genes from the 22,767 genes with expression data out of 23,042 total genes in the genome (Ensembl.org Assembly; AstMex102; Genebuild last updated July 2016 [31, 43–45]). The number 500 was chosen because we used 493 SFARI genes in our analysis. Of this random sampling of a subset of genes, our results indicated that 48.0 ± 4.2% (mean ± 95% confidence interval) had a significant age × morph interaction, and 49.0 ± 4.4% were significantly differentially expressed between cave and surface fish at 72 hpf (Table 1). In contrast, we found that 61.1 and 60.7% of Category 1 and 2 genes, respectively, exhibited a significant age × morph interaction and 75 and 64.3% of Category 1 and 2 genes, respectively, were differentially expressed at 72 hpf. This result indicates that cavefish orthologs of human genes in Category 1 and 2 ASD-risk genes are enriched for differential expression between cave and surface fish (> 99.9 percentile of bootstrapping probability).

To evaluate whether this observed gene enrichment is specific to ASD or applicable to other psychiatric diseases, we also examined genes involved in schizophrenia (SCZ), which shares many symptoms with cavefish and ASD [46]. One database for SCZ-risk genes lists 44 genes as being tightly associated with the disease, and another more recent database contains 304 genes (www.szgene.org and www.szdb.org, respectively). Unlike ASD-risk genes, these SCZ-risk genes do not show enrichment for differential expression between cavefish and surface fish compared with the random sampling of gene subsets (of the SCZ-risk genes in szdb.org, 39.8 to 55.2% show significantly different expression between cavefish and surface fish, Additional file 3). Thus, differential gene expression between cavefish and surface fish appears to have more similarities to ASD than SCZ.

The ASD-risk genes are included in the SFARI database mainly based on genetic association studies (evaluation of genetic variation in human cohorts), in which the expression direction—down or up regulation—is not taken into account [30, 45, 47]. The specific direction of expression of genes in a pathway or network can provide evidence as to whether these molecular pathways are strengthened or attenuated (co-expression network) [33, 35, 48]. To address this, we compared the direction of gene expression (up or down) between cavefish versus surface fish with that observed in ASD patients versus controls, by utilizing the human brain transcriptome of post-mortem ASD patients [33–35]. Of the 58 and 3442 human orthologs that exhibited significantly different gene expression between ASD patients and controls in two different studies that both use postmortem cortices (Voineagu et al. 2011 and Parikshak et al., 2016 respectively) [33–35], 74.1 to 74.6% were also differentially expressed between cavefish and surface fish (Table 2, Additional file 4).

**Table 2:** Direction of gene expression (up- or down-regulated) in this and previously published studies (cavefish compared with surface fish, and cases compared with controls)

	# orthologs of SFARI Gene that express differently (up or down)	Transcriptome from the cortices of ASD patients (Voineagu et al., 2011)	Transcriptomes from multiple tissues of ASD patients (review: Ansel et al., 2017)	Transcriptome from the cortices of ASD patients (Parikshak et al., 2016)
*A. mexicanus* (Whole Embryo 10–72 hpf) Reanalyzed in this study	335 of 409† (81.9%)	43‡ of 58 orthologs expressed differently between CF and SF (74.1%)	51‡§ of 77 orthologs expressed differently between CF and SF (66.2%)	2567‡ of 3442 orthologs expressed differently between CF and SF (74.6%)
		31 of 51‡ genes expressed in the same direction: (60.7%)	27 of 45‡§ directionally expressed genes are in the same direction: (60.0%)	1843 of 3152‡ directionally expressed genes are in the same direction: (58.5%)
BTBR Mouse (Hippocampus) Provenzano et al., 2016	30 of 493 (6.1%)	2 of 65 orthologs expressed differently in BTBR mouse (3.0%)^a^	9§ of 105 orthologs expressed differently in BTBR mouse (8.6%)^b^	216 of 4042 orthologs expressed differently in BTBR mouse (5.3%)^c^
		0 of 2 genes expressed in the same direction (0.0%)	4 of 7§ directionally expressed genes are in the same direction (57.1%)	109 of 216 directionally expressed genes are in the same direction (50.5%)
ASD patients (Blood) Pramparo et al., 2015	51 of 493 (10.3%)	11 of 65 human genes expressed differently in ASD patient’s blood (16.7%)^d^	16§ of 107 human genes expressed differently in ASD patient’s blood (14.8%)^e^	485 of 4425 human genes expressed differently in ASD patient’s blood (11.0%)^f^
		7 of 11 genes expressed in the same direction (63.6%)	4 of 7§ directionally expressed genes are in the same direction (57.1%)	179 of 485 directionally expressed genes are in the same direction (36.9%)
Neural cells derived from iPS cell of ASD patient Mariani et al., 2015	95 of 493 (19.3%)	18 of 65 of human genes expressed differently in neurons derived from iPS cells of ASD patients (27.7%)^g^	21§ of 107 human genes expressed differently in neurons derived from iPS cells of ASD patients (19.6%)^h^	527 of 4425 human genes expressed differently in neurons derived from iPS cells of ASD patients (11.9%)^i^
		0 of 18 human genes expressed in the same direction (0%)	1 of 16§ directionally expressed genes are in the same direction (6.3%)	163 of 527 directionally expressed genes are in the same direction (30.9%)

† 84 of 493 orthologs were not found in the *Astyanax* gene build at 2016 (Ensembl.org Assembly: AstMex102; Genebuild at Jul 2016)

‡ Some orthologs of human genes have multiple paralogs in *A. mexianus* (i.e. *shank3a* and *shank3b*)

§ We excluded genes that showed inconsistent expression directions between multiple reports (i.e. up-regulated in one paper but down-regulated in another [34])

Χ^2^ tests for differentially expressed genes against total genes between *A. mexicanus* and BTBR Mouse (^a^: Χ^2^ = 30.2, *P* = 1.17 × 10^− 7^; ^b^: Χ^2^ = 31.3, *P* = 6.57 × 10^− 8^; and ^c^: Χ^2^ = 1785.6, *P* < 1.0 × 10^− 10^), between *A. mexicanus* and patients’ blood (^d^: Χ^2^ = 14.9, *P* = 3.48 × 10^− 4^; ^e^: Χ^2^ = 21.7, *P* = 9.40 × 10^− 6^; and ^f^: Χ^2^ = 1445.5, *P* < 1.0 × 10^− 10^), and between *A. mexicanus* and patients’ iPS cells (^g^: Χ^2^ = 8.1, *P* = 0.0136; ^h^: Χ^2^ = 16.3, *P* = 1.66 × 10^− 4^; and ^i^: Χ^2^ = 1377.2, *P* < 1.0 × 10^− 10^). All of these tests have df = 1. *P*-values were multiplied by the number of the tests (Bonferroni correction)

Among the differentially expressed genes, 58.5 to 60.7% of cavefish genes showed the same direction of expression (i.e. cavefish relative to surface) as the human genes from the ASD transcriptome studies (i.e. ASD patients relative to controls) (Table 2, Additional file 4) [33–35]. This includes the down-regulation of *distal-less homeobox 1* (*DLX1*) and the up-regulation of *bag family molecular chaperone regulator 3* (*BAG3*) and *chloride intracellular channel protein 1* (*CLIC1*) cavefish orthologs relative to surface fish, which consistently showed similar patterns of expression in humans with ASD relative to controls [33, 35].

In contrast to the high percentage of ASD orthologs that show differential gene expression between cavefish and surface fish (Table 2), other ASD models—including a classic ASD mouse model (BTBR mouse) [49], the blood cells of ASD patients [50], and the neurons derived from induced pluripotent stem cell (iPS cell) of ASD patients [51]—exhibited much lower concordance with human brain ASD transcriptomic studies (Table 2). For several orthologs of Category 1 SFARI genes, we also observed expression differences between the brains of surface fish and cavefish by quantitative RT-PCR (Additional file 5) in later developmental stages (from 1 month to 1 year old). Overall, these transcriptomic analyses indicate that *A. mexicanus* cavefish and humans with ASD share similar patterns of ASD-risk gene expressions that could underlie shared ASD-like behaviors.

To survey additional parallels, we examined patterns of molecular evolution in DNA sequences of ASD-risk genes. We tested whether ASD-risk genes in *A. mexicanus* are highly divergent (i.e., potentially under positive selection) between cavefish and surface fish [52]. We identified the number of genes within the ASD-risk genes that are divergence outliers between cavefish and surface fish based on three metrics from population genetics: (i) top 5% of *F*_ST_ for all genes across the genome (indicates the difference in allele frequencies between populations), (ii) top 20% of *D*_XY_ (pairwise nucleotide differences between two populations) and/or (iii) *P*-value < 0.05 for hapFLK [53] (detects selection signatures based on population haplotype frequencies and is more an explicit test for positive selection, where as *F*_ST_ and *D*_XY_ are measures of divergence) (Table 3, Additional files 6 and 7). Since hapFLK is an explicit test of selection, we only used hapFLK to test for enrichment of ASD-risk genes for positive selection relative to the genome as a whole.

**Table 3:** Gene set enrichment analysis based on Fisher’s exact test with Yate’s continuity correction

Divergence metrics	Comparisons	Significant ASD genes	Total ASD genes	Significant genes in genome	Total genes in genome	Yate’s Chi Square X^2^	Degrees of freedom	Yate’s *P*-value
*hapFLK*	Whole genome	86	635^a^	1648	22,710	34.557	1	< 0.0001

Gene enrichment for ASD genes was based on comparisons between Choy surface (Choy) and Cave (Pachón). Calculations were performed using the *chisq.test* function in R

^a^Note that total ASD genes for hapFLK is lower than the total for orthologs and paralogs due to missing data

We tested whether the cavefish orthologs of 493 ASD-risk genes (SFARI gene Category 1–4 and Category S) were enriched for genes identified to be under positive selection via hapFLK. We included any *Astyanax* paralogs for ASD-risk genes (see Additional file 7), and used a Fisher’s exact test with Yate’s correction [54] to test whether the number of ASD-risk genes with *P*-values < 0.05 via hapFLK was overrepresented relative to total number of genes in the genome with P-values < 0.05 via hapFLK.

We found that the ASD-risk genes are enriched (13.5%) relative to all the genes in the genome (7.3%) for signatures of selection using haplotype frequency (hapFLK, *P* <  0.05; Table 3, Additional file 7). Thus, ASD-risk genes were ~ 2× more likely to exhibit a signature of positive selection than were the genes in the genome generally (odds-ratio 1.94; 95% confidence interval: 1.54–2.45). ASD-risk genes in humans are also hypothesized to be enriched for signatures of positive selection [55].

Since we needed to set the cut-offs for *F*_ST_ and *D*_XY_ measures to percentages of the genome, enrichment tests were not logical, and we performed Kruskal-Wallis tests to assess differences between the ASD gene set compared to all genes in the *Astyanax mexicanus* genome for *F*_ST_ and *D*_XY_. For all comparisons, genes with no data were removed. Note that hapFLK is based on cave and surface comparisons across 45 *Astyanax* samples from five populations (see Methods), which included additional populations that are beyond the scope of the work here. *F*_ST_ and *D*_XY_, however, were only focused on comparisons between Pachón cave population and Choy surface population.

Although we documented that the ASD-risk genes were enriched for significant hapFLK tests relative to the genome, we found that of the 661 Category S and Categories 1–4 SFARI genes with sequence data_,_ 19 were divergence outliers for *F*_ST_ (cut-off top 5% of all genes across the genome; Additional file 7; seven of these were divergence outliers with multiple metrics) and 51 were divergence outliers for *D*_XY_ (cut-off top 20%; 13 of these were divergence outliers defined by multiple metrics; Additional file 7), which are both a lower number of genes than what would be expected solely from our percent cut-off metrics (Note: *F*_ST_ and *D*_XY_ plots for divergence outliers which meet at least two metrics are shown in Additional file 6). ASD-risk genes that pass our outlier threshold for *F*_ST_ (top 5% of all genes genome-wide) are on average no more divergent between cavefish and surface fish than genome-wide non-ASD-risk genes that also passed the outlier threshold *F*_ST_ (top 5%) (*F*_ST:_ Kruskal-Wallis test; X^2^ = 1.727, df = 1, *P*-value = 0.189, Additional files 6 and 7). When comparing genes that did not pass our *F*_ST_ threshold (i.e., the bottom 95% of *F*_ST_ values across the genome), ASD-risk genes were significantly more divergent than non-outlier, non-ASD-risk genes, but the effect size was small (mean ASD-risk genes = 0.20, mean non-ASD-risk genes = 0.23, *F*_ST:_ Kruskal-Wallis test; X^2^ = 4.781, df = 1, *P*-value = 0.029, Additional files 6 and 7). Thus, we have little evidence that ASD-risk genes are on average more divergent for *F*_ST_ than non-ASD-risk genes.

Interestingly, we found evidence that divergence outlier ASD-risk genes for *D*_XY_ were more conserved than divergence outlier non-ASD genes. *D*_XY_ outlier ASD-risk genes were ~ 0.5× less divergent between cave and surface fish than outlier non-ASD-risk genes (*D*_XY:_ Kruskal-Wallis test; X^2^ = 29.285, df = 1, *P*-value < 0.001, Additional file 6). These results suggest that ASD-risk genes, which fall within our outlier cut-offs for *D*_XY,_ may be experiencing purifying selection relative to outlier genes in the rest of the genome. In contrast, non-outlier ASD-risk genes and non-outlier, non-ASD-risk genes in the remainder of the genome do not differ in their level of divergence for *D*_XY_ (*D*_XY:_ Kruskal-Wallis test; X^2^ = 0.046, df = 1, *P*-value = 0.830, Additional files 6 and 7). Considering that many ASD-risk genes in human were found as constrained [56, 57], this finding indicates another similarity between cavefish and human in the evolution of ASD-genes.

Analysis with Ingenuity Pathway Analysis Comparison Analysis (IPA) [48] highlights a further potential relationship between the evolution of ASD-risk genes and cavefish traits (Additional file 8). While many functional categories are enriched for non-outlier and outlier ASD-risk genes, some functional categories are only enriched in outlier ASD-risk genes (Additional file 8). In comparison to non-outlier ASD-risk genes, outlier ASD-risk genes (defined by top 5% of *F*_ST,_ top 20% of *D*_XY,_ and/or significant hapFLK scores) are enriched for functions that include auditory disease, digestive system development and function, inflammatory diseases, lipid metabolism, ophthalmic disease, and others. These results were consistent even when we imposed a more stringent cut-off for *D*_XY_ (top 5%). Many of these functional categories have been observed as co-morbid symptoms with ASD [58, 59] (Additional file 8). These functional categories map well to phenotypes likely under selection in cavefish [20], as well as known symptoms in ASD patients.

**Fig. 1:**
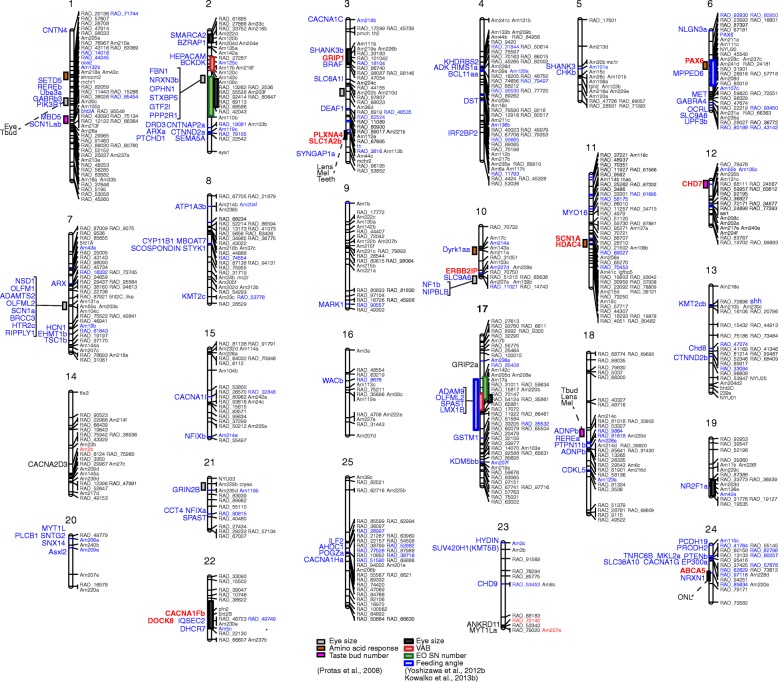
The congruency between quantitative trait loci and ASD-risk genes highlights potential genetic hubs for gene regulation. Linkage map constructed from 115 F2 hybrid progeny of a cross between a single surface fish female and a single male Pachón cavefish. The map includes 699 markers assembled into 25 linkage groups that collectively span 1835.5 cM. Colored bars represent approximate position of QTL for eye size, chemical (amino acid) sensing ability, taste bud number, VAB level, and the number of mechanosensory superficial neuromast at the eye orbit (EO SN) as indicated [74, 109]. Lens: lens size, Mel: melanophore number, Teeth: teeth number, Eye: eye size, Tbud: taste bud number, ONL: thickness in the outer nuclear layer of retina [110]. Each linkage group is annotated with genomic marker (right side) and anchored ASD-risk genes (left side). Blue characters in genomic markers are the ones that share the same genomic scaffold as the ASD-risk genes on the left side. Red characters in ASD-genes are the ones that show the signatures of divergence shown in Additional file 7. Other genes (at the left) are successfully anchored Category 1 and 2 SFARI Genes (Additional file 9, also Additional file 1)

Multiple cavefish orthologs of ASD-risk genes overlap with known quantitative trait locus (QTL) intervals for behavioral and sensory traits (Fig. 1, Additional file 9). The ASD-risk genes that are divergence outliers by one or more divergence metrics and are under previously mapped QTL are *abca5, cacna1fb, chd7, dock8, erbin* (i.e. *errbb2ip*)*, grip1, hdac4, pah, pax6, plxna4, scn1a, slc1a2b* (Fig. 1). Many of the divergent outlier genes are not under QTL, which may be because of the fragmented nature of the current genome sequence of *A. mexicanus*. However, this initial analysis revealed that many of the outlier genes under QTL for eye size, amino acid response, and taste bud number are members of two major gene networks that have been suggested to be involved in ASD: synaptic function (*cacna1fb, dock8, erbin, grip1, plxna4, scn1a, slc1a2b*) and epigenetic regulation (*chd7, hdac4*) [23, 56, 60]. Considering that eye-size is associated with adherence behavior [19] and chemosensory organs can modify wakefulness [61], this result implies that some of the putatively selected genes in cavefish may also be associated with ASD-like behavioral phenotypes.

**Fig. 2:**
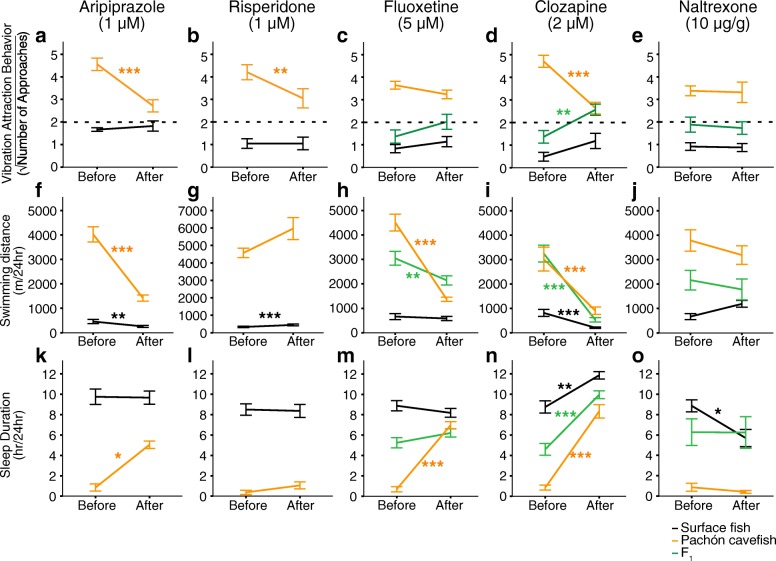
Human drugs for ASD mitigated cavefish-type symptoms in F_1_ hybrid and cavefish. (**a-e**) Adherence to 40-Hz vibration stimulus. Vibration attraction behavior is represented by the square-rooted number of approaches during a 3-min assay. (**f-j**) Swimming distance (m per 24-h assay). (**k-o**) The changes in sleep duration (h per 24-h assay). Before and after treatment of drugs used for ASD patients—aripiprazole (**a**, **f**, **k**), risperidone (**b**, **g**, **l**), fluoxetine (**c**, **h**, **m**), clozapine (**d**, **i**, **n**), naltrexone (**e**, **j**, **o**)—were observed for 24 h each and plotted with means ± s.e.m. In these cases, there are significant shifts of cavefish behaviors after treatment towards the surface fish behaviors before treatment, except for the naltrexone treatments. Stars indicate the significant behavioral changes between before and after drug treatments (paired *t*-test adjusted by Bonferroni correction, ***: *P* < 0.001, **: *P* < 0.01, *: *P* < 0.05). Black line: surface fish, and orange line: cavefish. All statistics are available in Additional file 11. Black dashed lines in a-e indicate the threshold level of vibration attraction behavior (square-rooted number of approaches equals 2) [20]

The results presented above indicate that the ASD-risk genes in cavefish and humans share evolutionary and gene expression signatures. Additionally, we sought to understand if cavefish and ASD patients respond similarly to drugs used to treat ASD. Accordingly, we treated *A. mexicanus* with the U.S. Food and Drug Administration-approved ASD drugs, aripiprazole and risperidone, and classic antipsychotic drug, clozapine [62, 63]. These drugs act as agonists and/or antagonists of multiple receptors for the neurotransmitters dopamine, serotonin, histamine, adrenalin/noradrenalin, and/or acetylcholine [62, 63]. We also treated selective serotonin reuptake inhibitor, fluoxetine, and an opioid blocker, naltrexone, which are used for ASD patients under the physicians’ direction [62–64]. We found that treating cavefish with aripiprazole, risperidone, clozapine and fluoxetine mitigated ASD-like behaviors in cavefish. The drugs significantly reduced adherence to a particular vibration stimulus (Fig. 2a, b, d), significantly reduced hyperactivity (swimming distance in Fig. 2f, h, i) and increased sleep duration (Fig. 2k, m, n) (see Additional file 10), which are similar to the responses observed in ASD-patients [62–64]. In contrast, these drugs showed little effect on surface fish behaviors (Fig. 2). The drug naltrexone, an opioid blocker that can mitigate hyperactivity and restlessness but not the core symptoms of ASD and did not change cavefish behaviors (Fig. 2e, j, o) [64]. These pharmacological studies indicate that cavefish may share similar neural pathways with ASD patients since chemical intervention alters similar behaviors.

## Discussion

For decades, evolutionary biologists have been interested in understanding how animals evolve multiple behaviors whose genetic bases are frequently independent and complex. Here we show that cavefish and ASD patients exhibit similarities in expression direction among ASD-risk genes, evolutionary signatures for ASD-risk genes, and responses to ASD symptom-treating drugs. These overlaps may indicate potential utilization of a suite of genes (ASD-risk genes) for the evolution of ASD-like behaviors in both human and cavefish. These ASD-risk genes are also known to be involved in social behavior of honeybees and mammals [5], suggesting that animals may frequently modify ASD-risk genes in the evolution of behaviors.

Modifications to most ASD-risk genes may not result in acute deleterious effects (except for some ASD-risk genes contributing to core neural activities that are often seen in de novo mutation) [23, 65, 66]. Indeed, in humans, many of the common variants for ASD-risk genes have small effects that might modify brain systems more subtly; in some cases, these modifications may even provide rather small beneficial effects [55, 67]. Accordingly, it has been reported that ASD positively correlates with childhood intelligence, college completion, and years of schooling; in addition, human ASD-risk genes show enrichment for signatures of positive selection [55]. Many of these small-effect alleles are thought to provide cumulative effects that can lead to ASD [68]. In contrast to these common small-effect variants, ASD-risk genes contributing to core neural activities are frequently found as rare alleles and/or de novo variants, and only a few of these variants are thought to be enough to promote ASD [21, 23]. In *A. mexicanus*, the quantitative trait loci (QTL) mapping of cavefish behaviors—adherence to vibration stimulus, loss of schooling and loss of sleep—showed small-effect sized QTL or no detectable QTL [20]. We therefore consider that cavefish is more similar to the common variant-induced ASD than ASD induced by rare or de novo variants. We expect that some of the cavefish ASD-risk genes under QTL may potentially yield a small beneficial effect in the cave environment (Fig. 1).

The IPA analysis revealed that, compared to non-selected ASD genes, positively selected and/or highly divergent ASD-risk genes in cavefish are enriched in the pathways of digestive system development/function, inflammatory diseases, lipid metabolism and energy metabolism. Indeed, some phenotypes are observed in cavefish relative to surface fish: cavefish exhibit fatty livers and insatiable appetite [69], lower metabolic rate than surface fish [70], higher infection susceptibility, and morphological change in gut (personal observations). These co-occurring symptoms raise a possible avenue for future work to explore how changes in immune function and metabolism (and perhaps gut function) influence a set of ASD-like behaviors in cavefish. Indeed, metabolism, immune and gut defects have been suggested in ASD etiology in humans [59, 71–73].

Notably, besides ASD-like behaviors, cavefish evolved eye degeneration, pigment-loss, widened jaws, an increase in fat tissue, an increase in number of teeth, and enhancement of non-visual sensory systems (mechanosensory lateral line, taste buds, and olfactory epithelium) [20, 69, 74–82]. Some of these cave-associated traits may be genetically correlated to ASD-like behaviors. For example, adherence to a particular vibration and loss of schooling showed significant correlations with eye size (vibration attraction behavior and the eye size, and schooling and eye size in F_2_ intercross: *r* = − 0.26 and *ρ* = 0.27, respectively [77, 83]). We also found that many of the positively selected orthologs of ASD-risk genes are under QTL intervals for eye size (Fig. 1). However, visual impairment itself may not induce a set of ASD-like behaviors. Rearing surface fish in the dark did not increase their vibration attraction behavior [19, 84]. Also, there is no correlation between loss of eyes and many of these behavioral traits [85]. Therefore, these data suggest that eye regression is not the major driver to shift a set of ASD-like behaviors. We then propose that, in a regard of the evolution of multiple behaviors, the eye regression has little contribution to ASD-like behaviors exhibited by the cavefish.

The potential for shared genetic underpinnings between cavefish and ASD can offer further insights into the etiology of ASD. Recent studies have helped clarify the genetics of de novo variants of ASD, which likely account for 3–15% of ASD cases [23, 56, 65, 86]. However, given that ASD is highly heritable and that all common and rare genetic variants are estimated to explain a significant proportion of ASD cases (17 ~ 50%) [23, 65, 66], an animal model for multiple heritable variants is still unavailable. Here, cavefish may serve to uncover gene-gene and gene-environment interactions, and to shed light on the effect of the gut-brain axis on ASD [20, 71].

## Conclusion

Overall, cavefish appears to be an advantageous platform upon which to untangle the polygenic evolutionary processes that generate a diverse behavioral spectrum in vertebrates. A recent study in honey bees—in which gene expression modifications between the brains of social and less-social honeybees were found to be enriched in ASD-risk genes—highlights further that ASD-risk genes for social behaviors are deeply conserved [5]. Above all, in many of animal species, including human and cavefish, a set of ASD-risk genes may impact the evolution of multiple behaviors [5].

## Methods

### Fish maintenance and rearing in the lab

*Astyanax mexicanus* surface fish used in this study were laboratory-raised descendants of original collections made in Balmorhea Springs State Park, Texas. Cavefish were laboratory-raised descendants originally collected from Cueva de El Pachón (Pachón cavefish) in Tamaulipas, Mexico.

Fish (surface fish and Pachón cave populations) were housed in the University of Hawai‘i at Mānoa *Astyanax* facility with temperatures set at 21 °C ± 0.5 °C for rearing, 24 °C ± 0.5 °C for behavior experiments, and 25 °C ± 0.5 °C for breeding [24, 87]. Lights were maintained on a 12:12 light:dark cycles [24, 87]. For rearing and behavior experiments, light intensity was maintained between 30 and 100 Lux. Fish husbandry was performed as previously described [20, 24, 87]. Fish were raised to adults and maintained in standard 42 L tanks in a custom-made water-flow tank system. Adult fish were fed a mixed diet to satiation three times daily starting 3 h after the lights came on (Zeitgeber time 3 or ZT3), ZT6 and ZT9 (TetraColor Tropical Fish Food Granules and TetraMin Tropical Fish Food Crisps, Tetra, Blacksburg, VA; Jumbo Mysis Shrimp, Hikari Sales U.S.A., Inc., Hayward, CA). All fish in the behavioral experiments were between 2.5–5 cm in standard length and between 6 and 18 months old. All fish care and experimental protocols are approved under IACUC (17–2560) at University of Hawai‘i at Mānoa.

### Genome survey and gene expression of ASD- and SCZ-risk genes

We identified a list of ASD-risk genes from the Simons Foundation Autism Research Initiative (https://sfari.org/resources/sfari-gene), which houses an extensive collection of data on genes potentially implicated in ASD in humans.

We queried the Simons Foundation Autism Research Initiative (https://www.sfari.org/resource/sfari-gene/; updated March 2017) databases and selected 493 ASD genes in Categories 1–4 and S (genes in each category are classified based on form of ASD, amount of risk conferred, and type of evidence for association with ASD, with higher categories indicating more evidence. ‘S’ category genes are associated not only with ASD but also with additional symptoms). For SCZ-risk genes, we queried the Schizophrenia Research Forum (http://www.szgene.org/; updated 2012) and (http://szdb.org/; updated May 2017) and extracted 44 genes in the ‘Top Results’ and 304 genes based on ‘Score 2–4,’ respectively. These were based on the evidence from human genome-wide association study, gene expression of postmortem brains and/or expression QTL) [45, 47]. For example, in SCZ genes, score 4 group includes the genes which meets 4 categories: (1) significantly differently expressed in patients, (2) significant in genome-wide association study, (3) significant in linkage and/or association study and (4) significant in pathway analysis (Additional file 3). Both of these gene sets were surveyed against the recent cavefish genome (Ensembl.org Assembly; AstMex102; Genebuild last updated July 2016 [31, 43–45]). First, human genes were queried with the Homologue (Orthologous Cavefish Genes) attributes to *Astyanax* genes in BioMart. Including the paralogs, we have a list of 677 *Astyanax* homologs of human ASD genes (Additional file 1). Similarly, we have a list of 766 homologs of human SCZ genes (Additional file 3).

For the RNAseq transcriptome analysis, variation in gene expression was analyzed using previously published RNAseq data (Genbank sequence read archive (SRA), accession code: PRJNA258661) [31, 38]. This dataset includes 50-pooled whole larvae from surface fish and Pachón cavefish (cave and surface fish pooled separately) at different developmental stages (10 h post fertilization (hpf), 24 hpf, 36 hpf and 72 hpf). Libraries for each pool of 50 larvae were prepared once and then sequenced on the Illumina HiSeq in three technical replicates [36, 88]. Data were analyzed by following previously published protocols [89]. Briefly, the exon information for *A. mexicanus* was acquired via the GTF file (Astyanax_mexicanus.AstMex102.89.gtf.gz) at ensembl.org (http://www.ensembl.org/info/data/ftp/index.html) and RNA sequences in fastq format were aligned to *A. mexicanus* genome sequence (“Astyanax_mexicanus.AstMex102.dna_sm.toplevel.fa” downloadable from ftp://ftp.ensembl.org/pub/release-91/fasta/astyanax_mexicanus/dna/) using STAR aligner version 2.5.1b [90]. First, we indexed the genome sequence for STAR by using “--runMode genomeGenerate” “--sjdbGTFfile. /Astyanax_mexicanus.AstMex102.84.gtf” “--genomeFastaFiles. /Astyanax_mexicanus.AstMex102.dna_sm.toplevel.fa”. We then mapped the raw fastq reads *to Astyanax* genome using “--outSAMtype BAM Unsorted.” After the alignment, a gene model database was built by the function *makeTxDbFromGFF* in the GenomicFeatures package (ver. 1.23.31) in R [91]. Once the database was built, we used the function *summarizeOverlaps* in the GenomicAlignments package (ver. 1.8.0 [91]) in R to adjust the read counts based on the exon information of each gene, which converted the read counts into FPKM (Fragments Per Kilobase Million). Expression levels were compared using the adjusted read counts.

To quantify expression differences between surface fish and Pachón cavefish at 72 hpf, we used the ‘*results*’ function in the DESeq package after estimating the data variance (ver. 1.12.0 [92]). We also tested the age × population interactions by setting the parameter ‘reduced = ~ population + age’ in the function of DESeq, followed by the ‘results’ function to extract the statistics [89]. All scripts have been made available on GitHub (https://github.com/masa-yoshizawa/Asty-RNAseq). The analysis at 72hpf was selected because (i) differences in expression patterns through developmental stages could affect the nervous system development (age × population interactions), and (ii) fish start moving/swimming according to the sensory inputs so that the neural wirings likely are being elaborated (comparable to infants: 72 hpf). Benjamini-Hochberg adjusted *P*-values and log_2_ expression differences between cavefish and surface fish were used to determine significance (as described in [89]). We performed bootstrapping using 9999 random samplings of 500 genes from the 22,767 genes with expression data out of 23,042 total genes in the genome of *A. mexicanus* (Ensembl.org Assembly; AstMex102; Genebuild last updated July 2016 [31, 43–45]). The number of 500 is chosen because we used 493 SFARI genes in our analysis (Table 1 and Additional file 3: S2).

### Population genomics and selection pressure analyses

#### Sample collection and preparation

All fin-clips of fish in the wild were collected under Mexico’s National Aquaculture and Fishing Commission (CONAPESCA) permit PPF/DGOPA - 106/2013 to Dr. Claudia Patricia Ornelas García and Mexico’s Secretariat of Environment and Natural Resources (SEMARNAT) permit 02241 to Dr. Ernesto Maldonado. Briefly, we included a core set of samples which contained the following: Pachón cave, *N* = 10 (9 newly re-sequenced + the reference reads mapped back to the reference genome) and surface (Río Choy), *N* = 9. After DNA extraction with Genomic-Tip Tissue Midi kits and DNeasy Blood and Tissue kit (Qiagen), individual samples were barcoded and next-generation sequencing libraries were prepped with Illumina TruSeq Nano DNA Sample Prep Kit (v3 reagents). Every five barcoded samples were pooled and sequenced in two lanes of the Illumina HiSeq2000 using 100 bp paired-end reads. Alignments of Illumina data to the *A. mexicanus* genome ver. 1.0.2 [31] were created with the BWA-mem algorithm in *bwa-0.7.1* [93]. The Genome Analysis Toolkit v.3.3.0 (GATK) and Picard v1.83 (http://broadinstitute.github.io/picard/) were used to filter alignments in accord with GATK Best Practices [94]. Hard filters were applied separately to SNPs and indels/mixed sites using the VariantFiltration and SelectVariants tools to remove low confidence calls from the dataset. Extensive details of sample collection and population genomic analyses are provided in (Herman et al. *submitted*) which includes additional samples. Samples used in the analyses presented here were submitted to the Project Accession Number: SRP046999 [38].

We employed multiple measures to identify regions of exceptional genomic divergence between cavefish and surface fish populations and to identify regions potentially under positive selection in the cavefish. For all population genomic measures, we excluded masked repetitive elements, indels (if present in any of the core set of samples), and 10 bp surrounding the bases affected by each indel. We used the masking_coordinates.gz file available for the *A. mexicanus* genome v1.0.2 though NCBI genomes FTP and performed the following measures with GATK-processed data on a per-gene basis, unless otherwise noted. We focused on multiple population genomic statistical metrics *D*_XY_, *F*_ST_, and hapFLK. Specifically, we conducted an enrichment tests on the positively selected ASD-genes relative to the rest of the genome using only the results from hapFLK.

We defined ‘divergence outliers’ as genes that were among the top 5% across the genome for *F*_ST,_ the top 20% for *D*_XY_ (as this is a less sensitive measure than *F*_ST_) and/or exhibited significant *p*-values using the program hapFLK. The top 5% is commonly used as a cut-off in outlier analyses [95]. The cut-off for *D*_XY_ as the top 20% was chosen as a less stringent criteria than for *F*_ST_ because this metric is less sensitive to allele frequency shifts [52]. Across SFARI genes Category 1–4, only 35 genes out of 113 total outlier genes were defined as outliers solely by the criteria of being in the top 20% for *D*_XY,_ and these were mainly used in the IPA analysis. We also redid IPA analysis with 5% as the cut-off for *D*_XY_ and obtained similar results. These two metrics exhibit different sensitivities and assumptions (reviewed in [95]). For example, relative measures of divergence (e.g. *F*_ST_) [96] detect divergent regions between two populations, yet may also detect outliers in low diversity regions that are false positives. Thus, we interpreted any outliers defined by relative measures of divergence in the context of pairwise nucleotide diversity (i.e. Pi), which is a measure of diversity within the populations. Absolute measures of divergence (*D*_XY_) [52], which are not confounded by low diversity, are not as sensitive to biologically meaningful divergence as relative measures [52] and may lead to false negatives. Thus, we used a combination of evidence to understand the molecular evolution of ASD-genes.

To identify genes in the top 5% for *F*_ST_ and top 20% for *D*_XY,_ we performed dense rankings where each measure (e.g. Pi surface, Pi cave, *F*_ST_ and *D*_XY_) was ranked for each gene in the genome. The higher the ranking, the higher the value was for that measure with dense ranks (e.g. 0.02, 0.03, 0.04, 0.04 was ranked 1, 2, 3, 3). To avoid issues in regions of low diversity, we excluded genes that were the lowest ranked 500 genes for Pi in the surface population as these genes may represent regions of low recombination in the genome. In addition, to measures of absolute and relative divergence, we also implemented the program hapFLK, which focuses on differences of haplotype frequencies between populations (see below). For hapFLK, we focused on genes with at least one hapFLK *P*-value that was less than 0.05. Due to the use of multiple metrics (e.g. *D*_XY_, *F*_ST_, hapFLK), we classified a focal psychiatric disease-related gene as a divergence outlier if the gene met any of the criteria of top 5% of genes for *F*_ST_, the top 20% for *D*_XY_ and/or had one *P*-value < 0.05 for hapFLK within the gene (Table 3, Additional files 6 and 7).

#### Basic population genomic metrics

We used VCFtools [97] to calculate Pi and *F*_ST_ and custom python scripts to calculate these metrics on a per-gene basis. We identified the allele counts per population with VCFtools and used these for subsequent *D*_XY_ calculations. For all metrics, we only used sites that contained six or more individuals per population (see Additional file 7). For *F*_ST_ and *D*_XY_, we focused on comparisons between Pachón cavefish and surface fish (Río Choy population). The Río Choy surface fish represent the population closest in our population genomic sampling to the surface fish population from Texas used in the current study [98].

#### hapFLK

hapFLK is an explicit test for positive selection and detects changes in haplotype frequencies that exceed what is expected for genetic drift given a hierarchical population structure [53]. hapFLK may be robust against bottlenecks and migration, and in analyses of various selective sweep measures across regions of known sweeps in dogs, hapFLK detected every focal sweep [95]. We used hapFLK ver. 1.3 https://forge-dga.jouy.inra.fr/projects/hapflk [53] with 45 *Astyanax* samples from five total populations (6–10 individuals per population) and two additional outgroups and included the following parameters: 30 haplotype clusters (-K 30), 20 EM runs to fit the LD model (−nfit = 10), and unphased data. *P*-values were estimated by fitting a standard normal distribution genome wide in R (Table 3, Additional file 7) [53].

### Quantitative PCR

From the lab-reared individuals, we anesthetized (with 0.5 mg/ml of buffered MS222 in ice-cold water) four individuals each at 1 month, 2 months, and 4 months old and two individuals at 12 months old from both the surface and Pachón cavefish populations. Whole brains from each individual were then carefully dissected out in ice-cold PBS [99] and collected into a pre-chilled 1.5 ml-tube on dry ice. The brains of each were homogenized in 1 ml QIAzol Lysis Reagent (Qiagen, Valencia, CA) by using a Micro Tube Homogenizer System (Wilmad-LabGlass, Vineland, NJ). The total RNA extraction was performed by using the RNeasy Plus Universal kit (Qiagen) with an elution volume of 20 μl. RNA quality and quantity were determined based on electrophoresis and Qubit 3.0 Fluorometer system (Thermo Fisher Scientific, Waltham, MA), respectively. iScript gDNA Clear cDNA Synthesis kit was used to eliminate the carryover of genomic DNA, followed by synthesis of cDNA by using iScript Reverse Transcription Supermix for RT-qPCR (Bio-Rad Laboratories, Hercules, CA).

The quantitative RT-PCR for the genes associated with ASD was performed. Three housekeeping genes *eef2.1a*, *rsp18* and *b2m* were used to normalize the quantification cycle (Cq). Quantitative real-time PCR was performed on a CFX96 Touch Real-Time PCR Detection System (Bio-Rad Laboratories) using the SsoAdvanced Universal SYBR Green Supermix (Bio-Rad Laboratories). Cycling parameters were: 1 cycle of 95 °C for 15 s, and 40 cycles of 98 °C for 5 s and 60 °C for 30 s. After quantitative real-time PCR, the melt curve analysis was performed between 65 °C – 95 °C with 0.5 °C step. Duration of each step was 2–5 s. This identified the annealing temperature for each PCR product, which informs the target specificity of the PCR reaction by monitoring whether the single length of PCR products (i.e. a single sharp peak of melting curve) was amplified [100]. Measurements of gene expression at each developmental stage (1 month, 2 months, 4 months and 12 months old) were technically repeated three times by using three wells of a PCR plate. Geometric average of Cq_target_ was subtracted by the geometric average of three repeats of three housekeeping genes at the same developmental stage (Cq_reference_: *eef2.1a*, *rsp18* and *b2m*), yielding ΔCq. Relative expression (ΔΔCq) of each gene at each target tissue (i.e. brain of 1, 2, 4 or 12 months from surface fish or cavefish) was then calculated by subtracting the ΔCq of cavefish brain at 1 month old from ΔCq of target tissue (Additional file 5). The sequences for PCR primers are reported in Table 4.

**Table 4:** PCR primers used in quantitative RT-PCR study

Primer Name	Ensemble Gene ID	Forward Sequence	Reverse Sequence
*shank3b*	ENSAMXG00000009680	AGTATGACCCACGGCTAGAG	CGATCACATAATCACTGTAGGAGG
*shank3a*	ENSAMXG00000004290	CGAATTACACCAGCAGAAATCAG	CCTCAGTAGCTCCGAAAGAC
*adnp2a*	ENSAMXG00000006355	AGAGTCACTGGATGTGATTCAC	TTCTTGGTTCAAGTCGATGATCTC
*suv420h1*	ENSAMXG00000020652	AAATGAACACCAGGTTTCGAC	AGAAAGTGATGTCTGCTCCA
*kmt5b*	ENSAMXG00000017258	GGCTGATGATTGAAACAGAGAC	CATGTCGTCTTCCATTTACTACC
*pogza*	ENSAMXG00000020522	TATTACACGCGTATTTCAGGG	TAGACACAGATATCCACGAAGAG
*ptena*	ENSAMXG00000010994	CGGGACTACTTGATTCTAACTCTG	TACAACTTCACCTTAAAGTTCGGG
*scn2a*	ENSAMXG00000004964	TCTTCACCTACATCTTCATCCTG	CCAAAGACACATCTACAATGAGG
*b2m*	ENSAMXG00000011344	TTCACACCTCAGAAGAACGA	ACTGCATTCTCCATCTGGT
*eef2a.2*	ENSAMXG00000018020	TATCATTGAGGAGTCTGGAGAG	TGGGTCGGATTTCTTAATTGG
*rps18*	ENSAMXG00000007922	CCATCAAGGGTGTTGGTAGG	TGCATAATGGTCACCACCC

### Drug treatment

ASD drugs were selected according to clinical trials and practices [62, 63, 101, 102]. Drug concentrations were determined based on previous experiments in model species [103–105]. Fluoxetine (1.0–28.5 μM; Sigma-Aldrich, St. Louis, MO), clozapine (0.1–12.5 μM; Selleck, Houston, TX), naltrexone (5–10 μg/body g; Selleck), aripiprazole (1–5 μM; Selleck), or risperidone (1–5 μM; Selleck) were delivered via bath application with PBS solution in conditioned water (for fluoxetine), via 0.1% dimethylformamide in conditioned water (for clozapine, aripiprazole and risperidone), or via injection through the body cavity (for naltrexone; less than 20 μl with a 27G insulin syringe) (Table 5). Injections were performed under anesthesia using 66.7 μg/ml of MS-222 (Tricaine, Sigma-Aldrich, St. Louis, MO). For sleep and hyperactivity assays, fish were bath-treated or given an intraperitoneal injection with each drug at Zeitgeber Time 1 (ZT1). Information for each drug is reported in Table 5. We started video recordings right after the time of injection (ZT2) then recorded for 24 h to measure sleep and hyperactivity levels (see below). For adherence assays (vibration attraction behavior; VAB assay, see below), fish were treated with the focal drug for at least 16 h (overnight), and then subjected to a 3-min behavioral assay (see Table 5). F_1_ hybrids of surface fish and cavefish were also assayed under the treatment of fluoxetine, clozapine and naltrexone, however, we had technical difficulties in raising sufficient numbers of F_1_ hybrids to also test hybrids for the aripiprazole and risperidone treatments.

**Table 5:** Drug information used in this study

Drug Name	Commercial Name	Target	Application Method	References
Aripiprazole	Abilify	Partial agonist for the receptors of dopamine, serotonin and others	Bath (1–5 μM)	[62]
Risperidone	Risperdal	Antagonist for the receptors of dopamine, serotonin and others	Bath (1–5 μM)	[62, 63]
Fluoxetine	Prozac	Selective serotonin reuptake inhibitor (SSRI)	Bath (1.0–28.5 μM)	[62]
Clozapine	Clozaril	Antagonist for the receptors of dopamine, serotonin and others	Bath (0.1–12.5 μM)	[62, 63]
Naltrexone	Revia	Opiate antagonist	Injection (5–10 μg/g)	[63]

### Sleep and hyperactivity

Fish were recorded under non-drug treated-conditions in a custom-designed 10.0-L acrylic recording chamber (457.2 × 177.8 × 177.8 mm in length × width × height, respectively, with 6.4 mm thickness) with opaque partitions that allow for 5 individually housed fish per tank (each individual chamber is 88.9 × 177.8 × 177.8 mm). This setup is approximately the same as used in [24]. Briefly, the recording chamber was illuminated with a custom-designed IR LED source for 24 h and with a white LED light that was set on a 12 h on, 12 h off cycle (Lampux 12 V Flexible Waterproof 5050 LED Strip Lights-Daylight White, Lighting EVER, Las Vegas, NV). White light was turned on at 7:00 am and turned off at 7:00 pm every day. Behavior was recorded after 4–5 days of acclimation for 24 h beginning two hours after the light was turned on (Zeitgeber time 2). Videos were recorded at 15 frames/sec using a USB webcam that was fitted with a zoom lens (Macro 1.8/12.5-75 mm C-mount zoom lens, Kowa American Corp., Torrance, CA). An IR high-pass filter was placed between the camera and the lens to block visible light. Videos were captured by the software, Virtualdub (Version 1.10.4, http://www.virtualdub.org/) with x264vfw codec and were subsequently processed using SwisTrack (Version 4, https://en.wikibooks.org/wiki/SwisTrack). Water temperature was monitored throughout the recordings, and no detectable differences were observed during the light and dark periods (24.0 °C ± 0.5 °C) [24]. The visible light during behavior recordings was approximately 30–100 Lux. Tracking parameters for detection were set as follows: the detection was set to ‘subject brighter than background’ and brightness contrast from 20 to 255; current frame weight set to 15; video sample rate set to 15 frames/sec, and pixel smoothing was turned off. We monitored sleep, activity, and arousal threshold via previously established protocols in *A. mexicanus* [24]. Sleep state was analyzed according to a previous study [24, 106]. Data was subsequently processed using custom-written Perl scripts (v5.10.0, www.perl.org) and Excel macro (Microsoft, Redmond, WA).

### Adherence to a particular vibration stimulus

We assayed VAB as described previously [19, 77, 107]. Briefly, We have acclimated fish individuals for 4–5 days prior to the assay in the cylindrical assay chamber (325 ml glass dish, 10 cm diameter 5 cm high, VWR, Radnor, PA, USA) filled with conditioned water (pH 6.8–7.0; conductivity 600–800 μS). During the assays, vibration stimuli were created by using a glass rod that vibrated at 35 or 40 Hz. The number of approaches (NOA) to the vibrating rod was video-recorded during a 3-min period under infrared illumination and counted using ImageJ 1.50o software (NIH, Bethesda, MD, USA).

### Statistics

For statistical comparisons, we performed parametric tests including student’s *t*-tests and one-way or two-way ANOVAs to compare between surface and cavefish. We performed Levene’s equality of variance test and visually inspected the distribution of the data, to look for violations of the assumptions of equal variance and normality. If violations were detected, we transformed the data by applying square-root or log transformation, accordingly [108]. Post-hoc Dunnett *t*-tests and Bonferroni correction were used for understanding which contrasts were significant. Above calculations were conducted using IBM SPSS 24.0.0.1 software (IBM, Somers, NY, USA) and all statistical scores are available in Additional files 10 and 11 or figure legends.

## Additional files

Additional file 1: Gene expression changes in *A. mexicanus* orthologs of SFARI genes (Category 1 to 4 and Category S in in March 2017). (XLSX 217 kb)

Additional file 2: Gene expression plots of *A. mexicanus* ASD-risk genes listed in SFARI Gene. Many of *A. mexicanus* orthologs of ASD-risk genes *in* Category 1–4 and Category S had significantly different expression between surface fish and cavefish at the stages of 10, 24, 36 and 72 h post fertilization (hpf). (PDF 1187 kb)

Additional file 3: Little enrichment of the expression shifts between surface fish and cavefish in SCZ-risk genes listed in SZgene.org or szdb.org. (PDF 235 kb)

Additional file 4: The direction of gene regulation (up or down) seen in *A. mexicanus* with that seen in ASD patients versus controls. (XLSX 559 kb)

Additional file 5: Example expression patterns of ASD-risk genes in SFARI Gene Category 1. Quantitative RT-PCR showed similarities to ASD patients. (PDF 74 kb)

Additional file 6: *D*_XY_ and *F*_ST_ statistics and plots for the top outliers whose divergence metrics passed our threshold (i.e. top 5% for *F*_ST_, top 20% for *D*_XY_, and *P* <  0.05 for hapFLK). (PDF 139 kb)

Additional file 7: Divergence outliers within the SFARI ASD-risk genes between cavefish and surface fish based on three metrics (i.e. top 5% for *F*_ST_, top 20% for *D*_XY_, and *P* < 0.05 for hapFLK). (XLSX 466 kb)

Additional file 8: Ingenuity Pathway Analysis Comparison Analysis revealed enriched pathway categories in diversified SFARI genes in cavefish. (PDF 8084 kb)

Additional file 9: ASD genes under diversification/positive selection and under QTL intervals. (XLSX 71 kb)

Additional file 10: Human drugs for psychiatric disease mitigated cavefish-type symptoms in a dose-dependent manner. (PDF 93 kb)

Additional file 11: Statistical scores for Fig. 2. (PDF 45 kb)

## Abbreviations

ASD: Autism spectrum disorder
CF: Cavefish
IPA: Ingenuity Pathway Analysis
iPS cells: Induced pluripotent stem cell
QTL: Quantitative trait locus
RNAseq: Ribonucleic acid sequencing
RT-PCR: Reverse transcription polymerase chain reaction
SCZ: Schizophrenia
SF: Surface fish
SFARI: Simons Foundation Autism Research Initiative
VAB: Vibration attraction behavior
