# Supplementary material for: The evolution of a series of behavioral traits is associated with autism-risk genes in cavefish
Source: BMC Evol Biol. 2018 Jun 18;18:89. doi: 10.1186/s12862-018-1199-9 (PMC6004695; doi:10.1186/s12862-018-1199-9)
Supplement: Supplementary file 2 — Gene expression plots of A. mexicanus ASD-risk genes listed in SFARI Gene. Many of A. mexicanus orthologs of ASD-risk genes in Category 1–4 and Category S had significantly different expression between surface fish and cavefish at the stages of 10, 24, 36 and 72 h post fertilization (hpf). (PDF 1187 kb) [file 12862_2018_1199_MOESM2_ESM.pdf]

## SFARI Gene Category 1 (page 1 of 1)

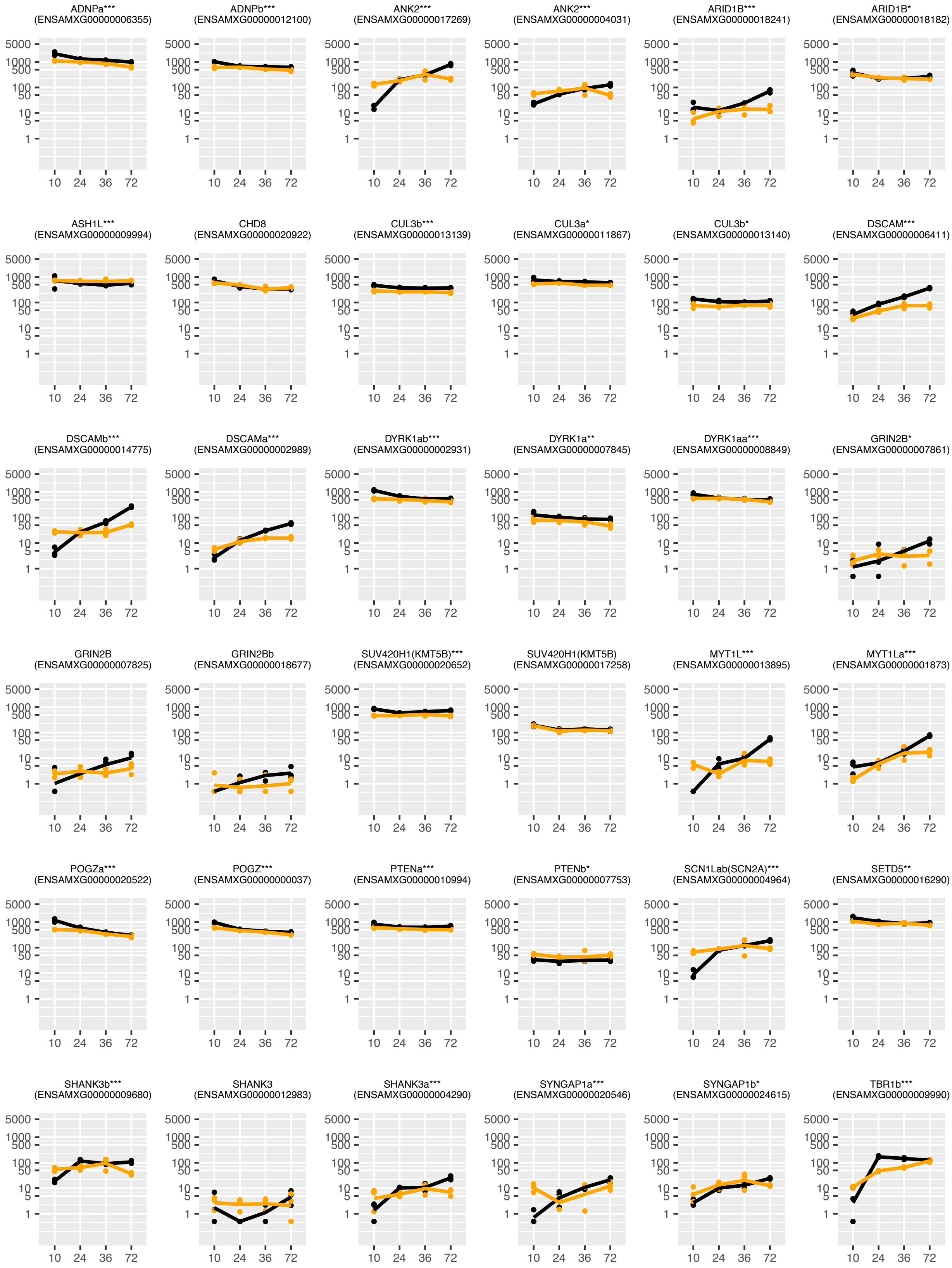

**Additional file 2.1—associated with Table 1. Many of the ASD-risk genes listed in SFARI Gene Category 1 had significantly different expression between surface fish and cavefish at the stages of 10, 24, 36 and 72 hours post fertilization (hpf).**
A set of 36 *A. mexicanus* genes (all paralogs of 18 out of 19 orthologs in Category 1) that are orthologs of human SFARI Gene Category 1 is shown (https://gene.sfari.org/autdb/GS_Home.do). Y-axis: normalized count of the number of sequence reads (Fragments Per Kilobase Million; FPKM). X-axis: hours post fertilization. Yellow dots and line represent the gene expression of cavefish, and the black dots and line represent that of surface fish. Each dot represents one of 3 replicates in each data point (morph and age). *: $P < 0.05$, **: $P < 0.01$, ***: $P < 0.001$ in the lower P-value between the age × morph interaction and the expression difference at 72 hpf after applying Benjamini-Hochberg correction[1]. See also Additional file 1.

# SFARI Gene Category 2 (page 1 of 2)

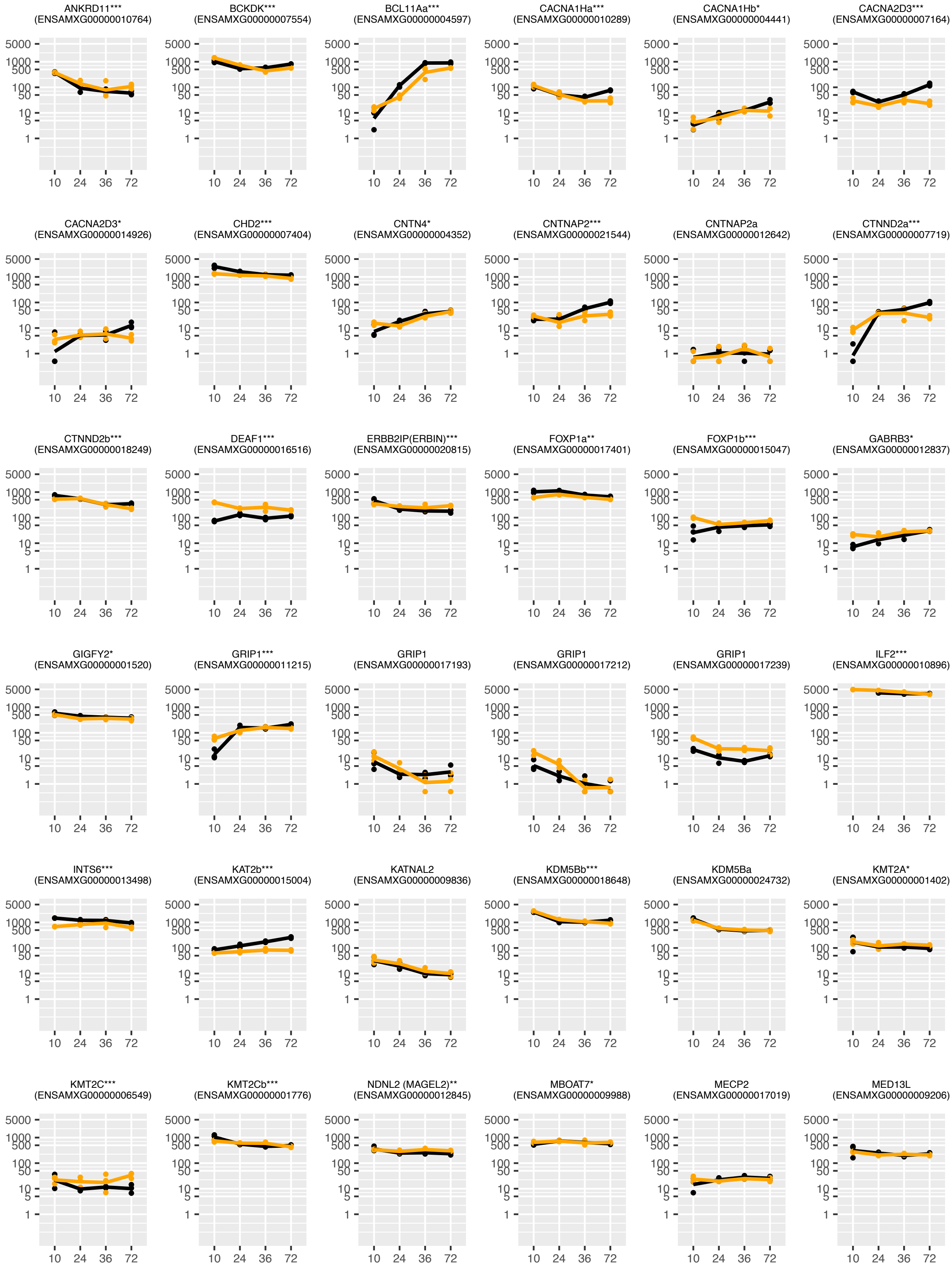

. SFARI Gene Category 2 (page 2 of 2)

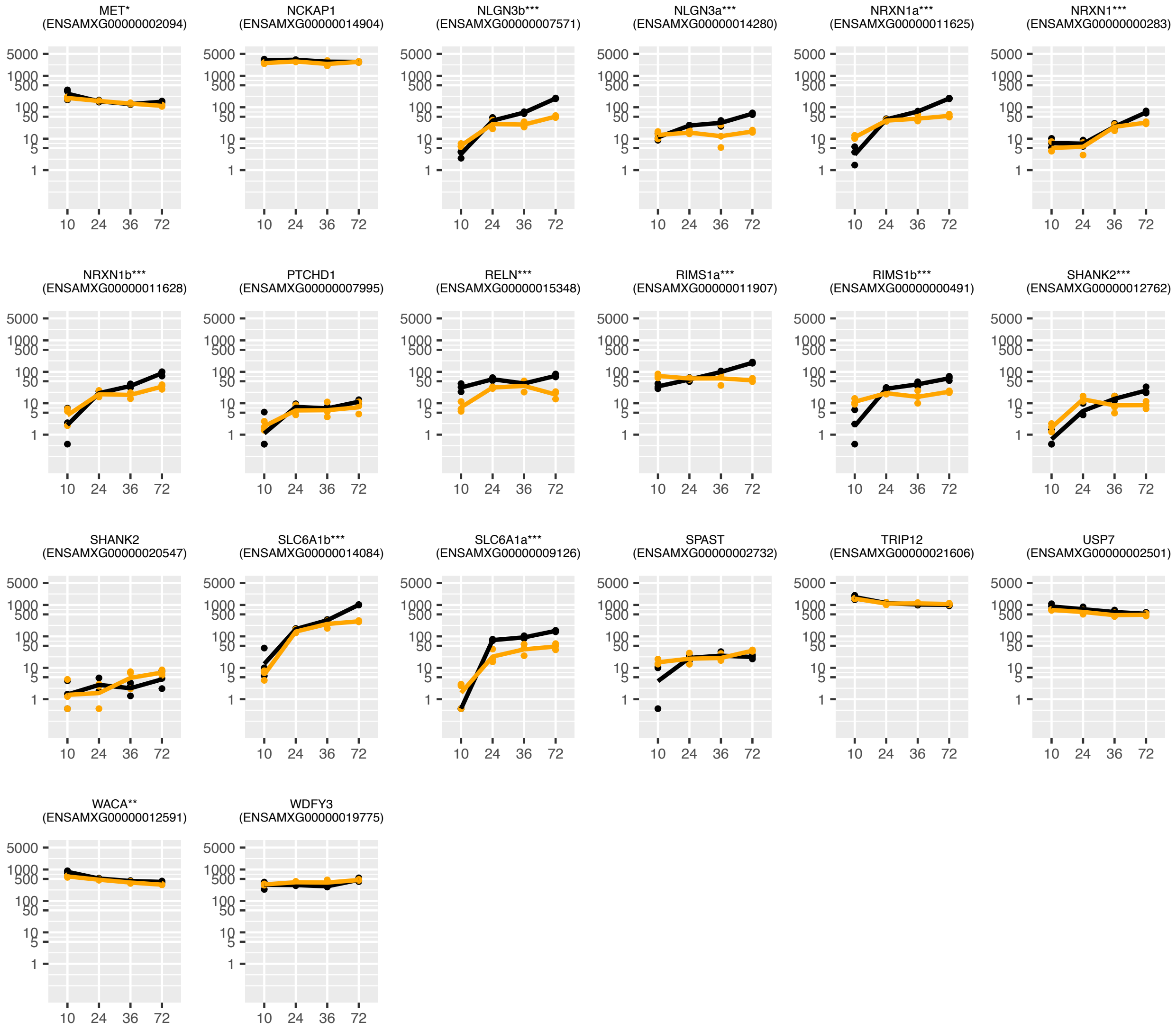


**Additional file 2.2—associated with Table 1. Many of the ASD-risk genes listed in SFARI Gene Category 2 had significantly different expression in surface fish and cavefish at the stages of 10, 24, 36 and 72 hours post fertilization (hpf).**

A set of 56 (all paralogs of 40 out of 43 orthologs in Category 2) *A. mexicanus* genes that are orthologs of human SFARI Gene Category 2 is shown (https://gene.sfari.org/autdb/GS_Home.do). Y-axis: normalized count of the number of sequence reads (Fragments Per Kilobase Million; FPKM). X-axis: hours post fertilization. Yellow dots and line represent the gene expression of cavefish, and the black dots and line represent that of surface fish. Each dot represents one of 3 replicates in each data point (morph and age). *: $P < 0.05$, **: $P < 0.01$, ***: $P < 0.001$ in the lower P-value of between the age × morph interaction and the expression difference at 72 hpf after applying Benjamini-Hochberg correction [1]. See also Additional file 1.

# SFARI Gene Category 1 (page 1 of 6)

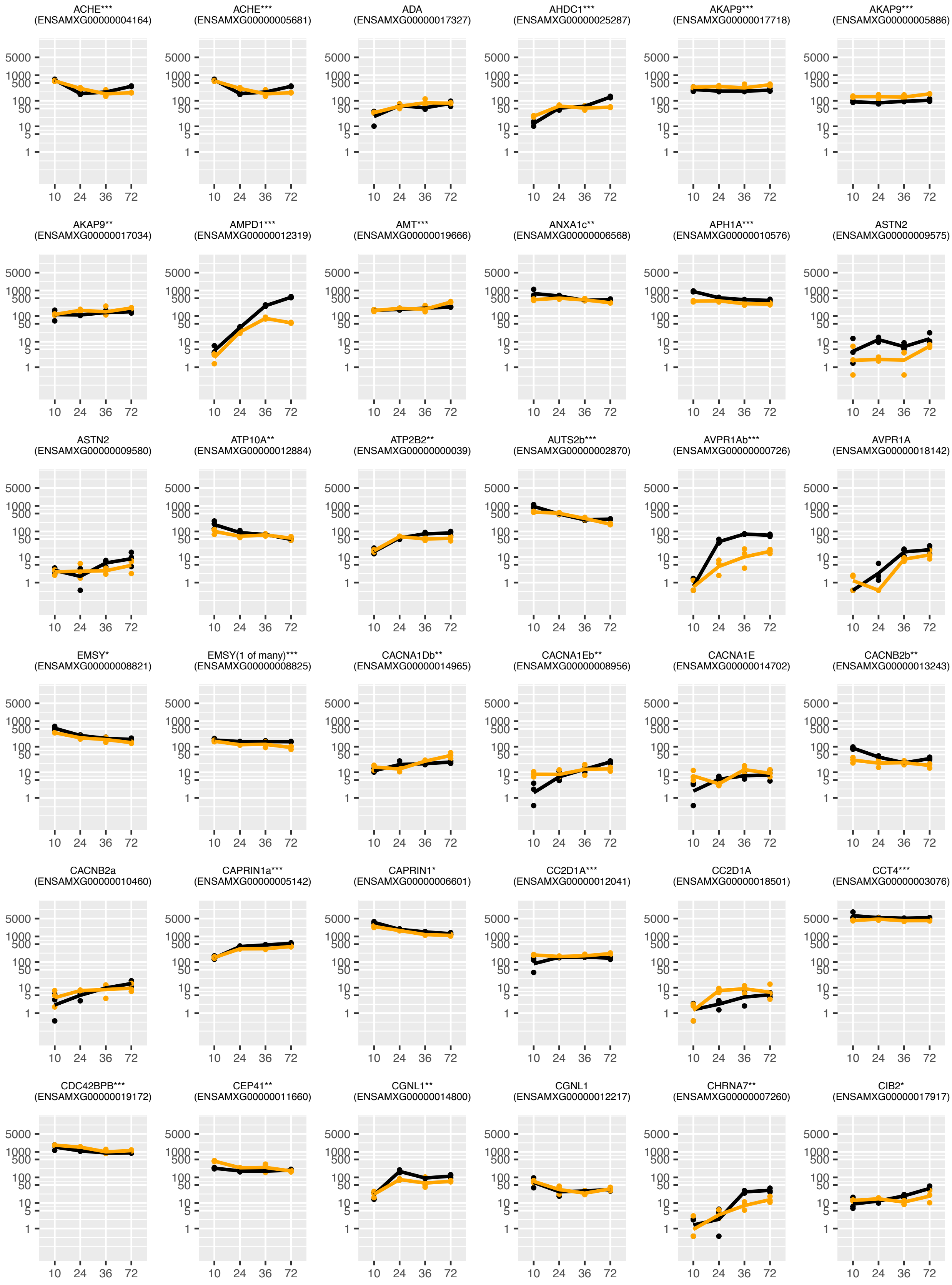

# SFARI Gene Category 3 (page 2 of 6)

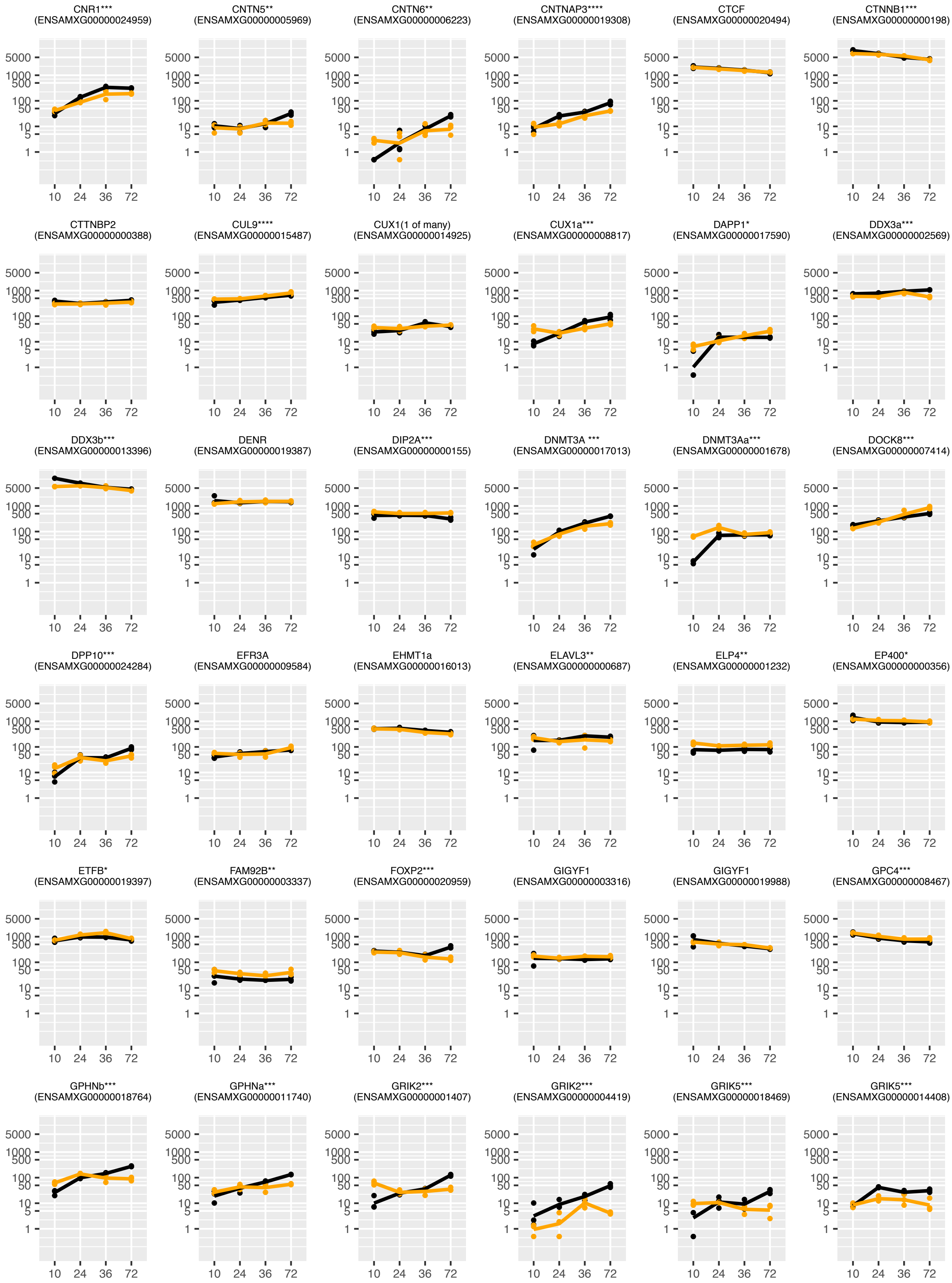

# SFARI Gene Category 3 (page 3 of 6)

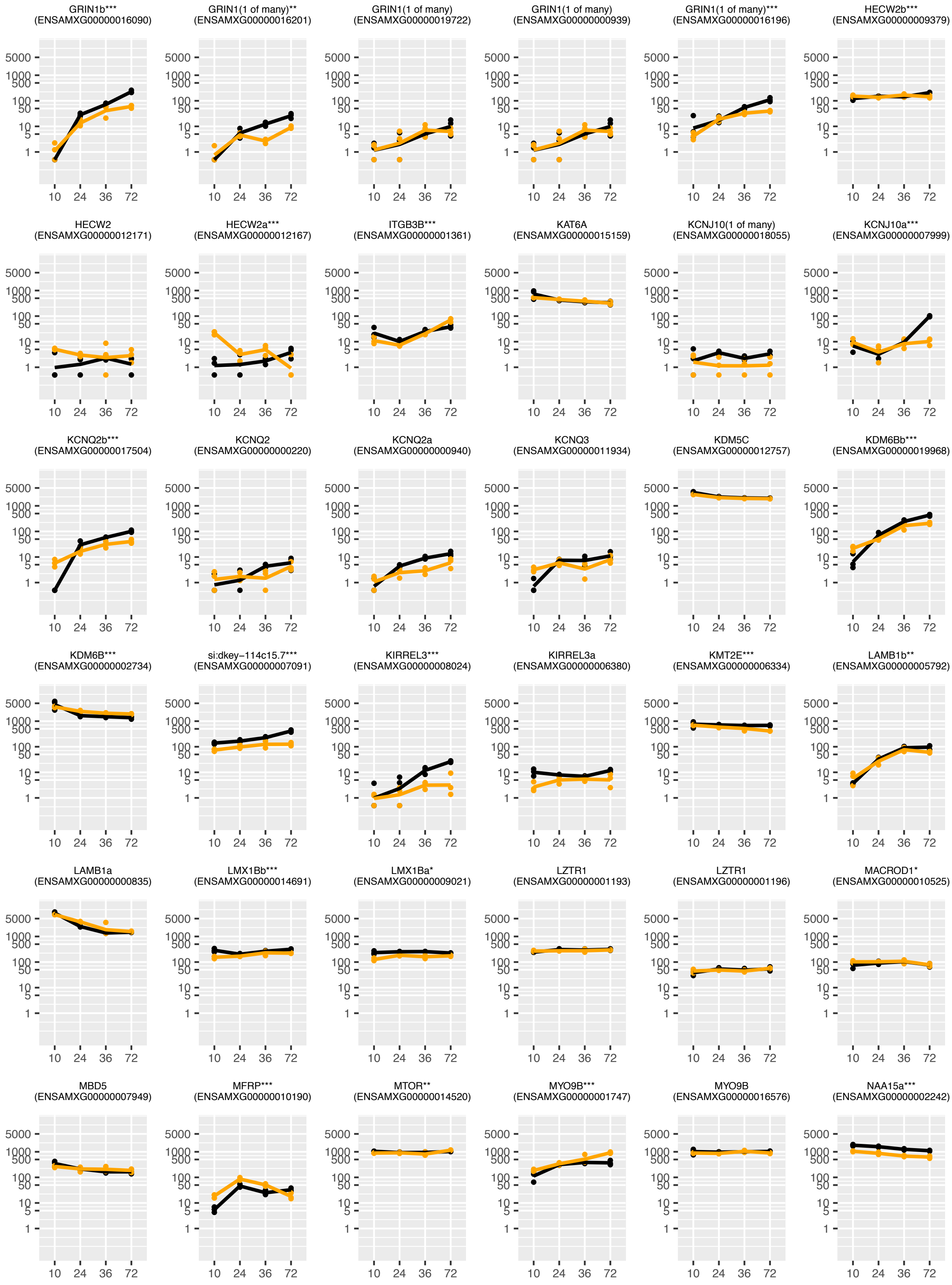

## SFARI Gene Category 3 (page 4 of 6)

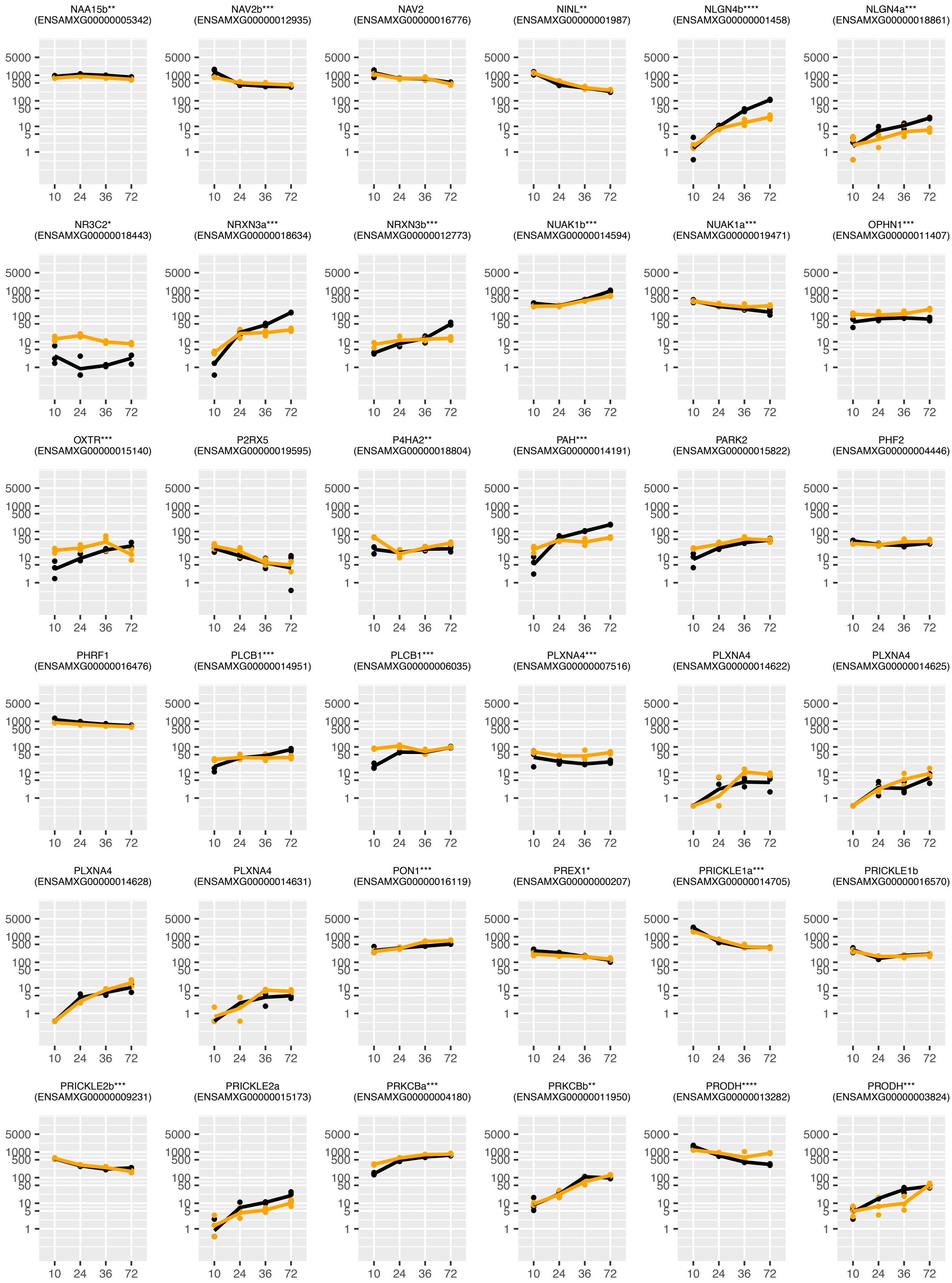

SFARI Gene Category 3 (page 5 of 6)

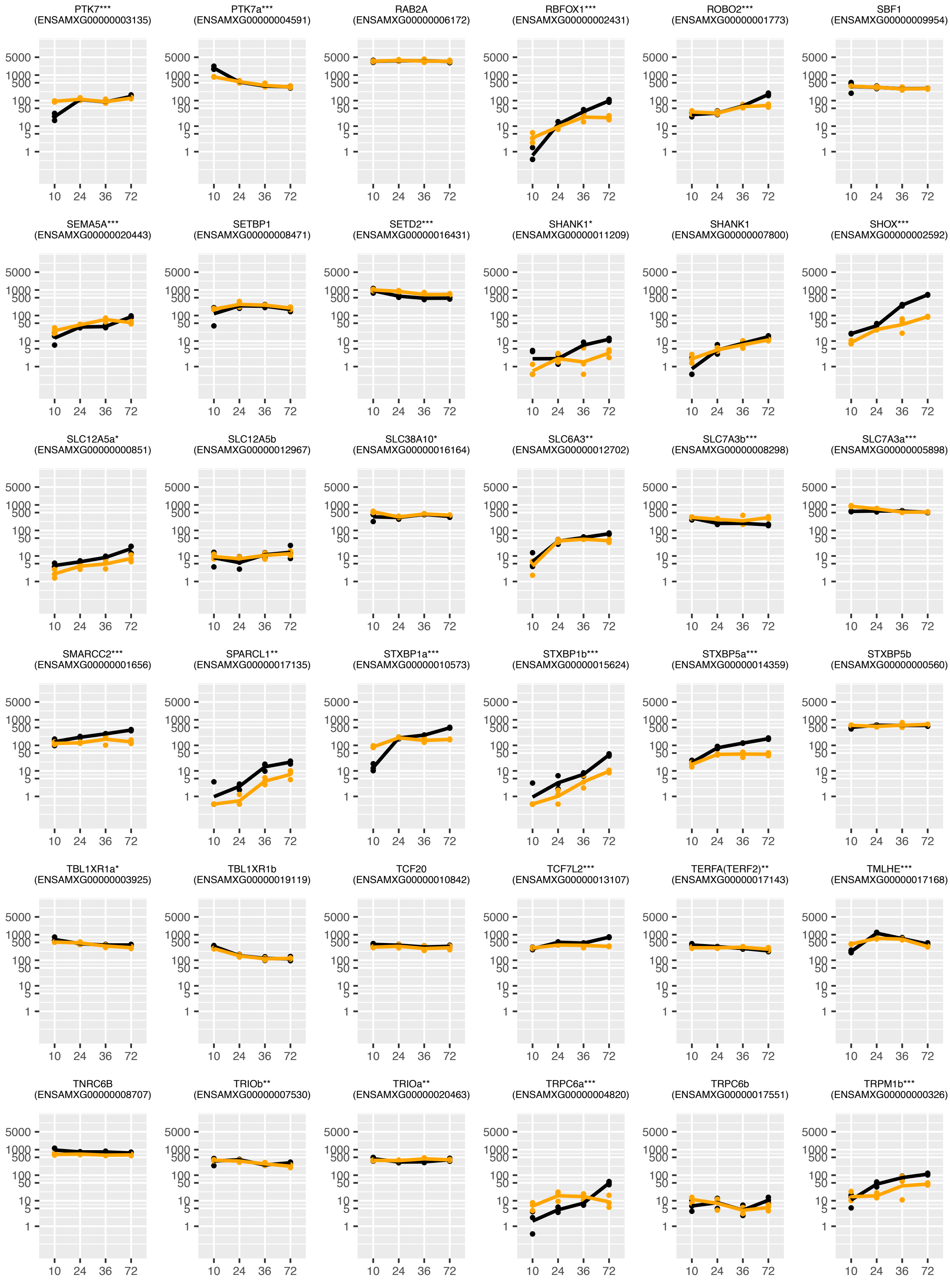

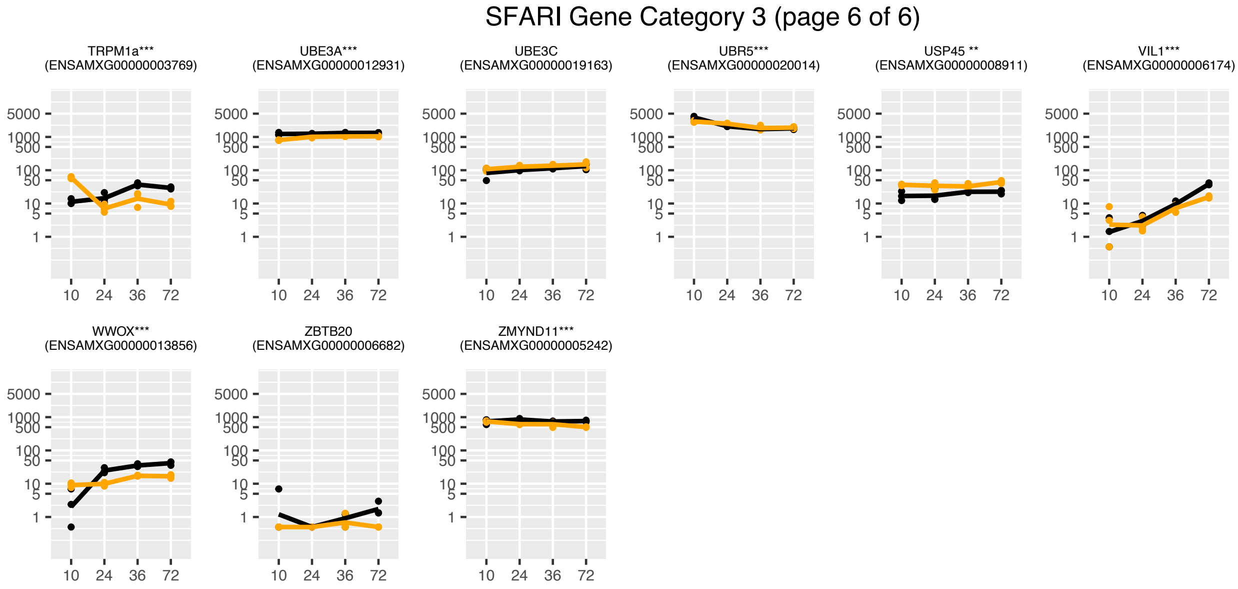


**Additional file 2.3—associated with Table 1. Many of the ASD-risk genes listed in SFARI Gene Category 3 had significantly different expression in surface fish and cavefish at the stages of 10, 24, 36 and 72 hours post fertilization (hpf).**
A set of 189 (all paralogs of 132 out of 139 orthologs in Category 3) *A. mexicanus* genes, orthologs of human SFARI Gene Category 3 is shown (https://gene.sfari.org/autdb/GS_Home.do). Y-axis: normalized count of the number of sequence reads (Fragments Per Kilobase Million; FPKM). X-axis: hours post fertilization. Yellow dots and line represent the gene expression of cavefish, and the black dots and line represent that of surface fish. Each dot represents one of 3 replicates in each data point (morph and age). *: $P < 0.05$, **: $P < 0.01$, ***: $P < 0.001$ in the lower P-value of between the age × morph interaction and the expression difference at 72 hpf after applying Benjamini-Hochberg correction [1]. See also Additional file 1.

## SFARI Gene Category 4 (page 1 of 9)

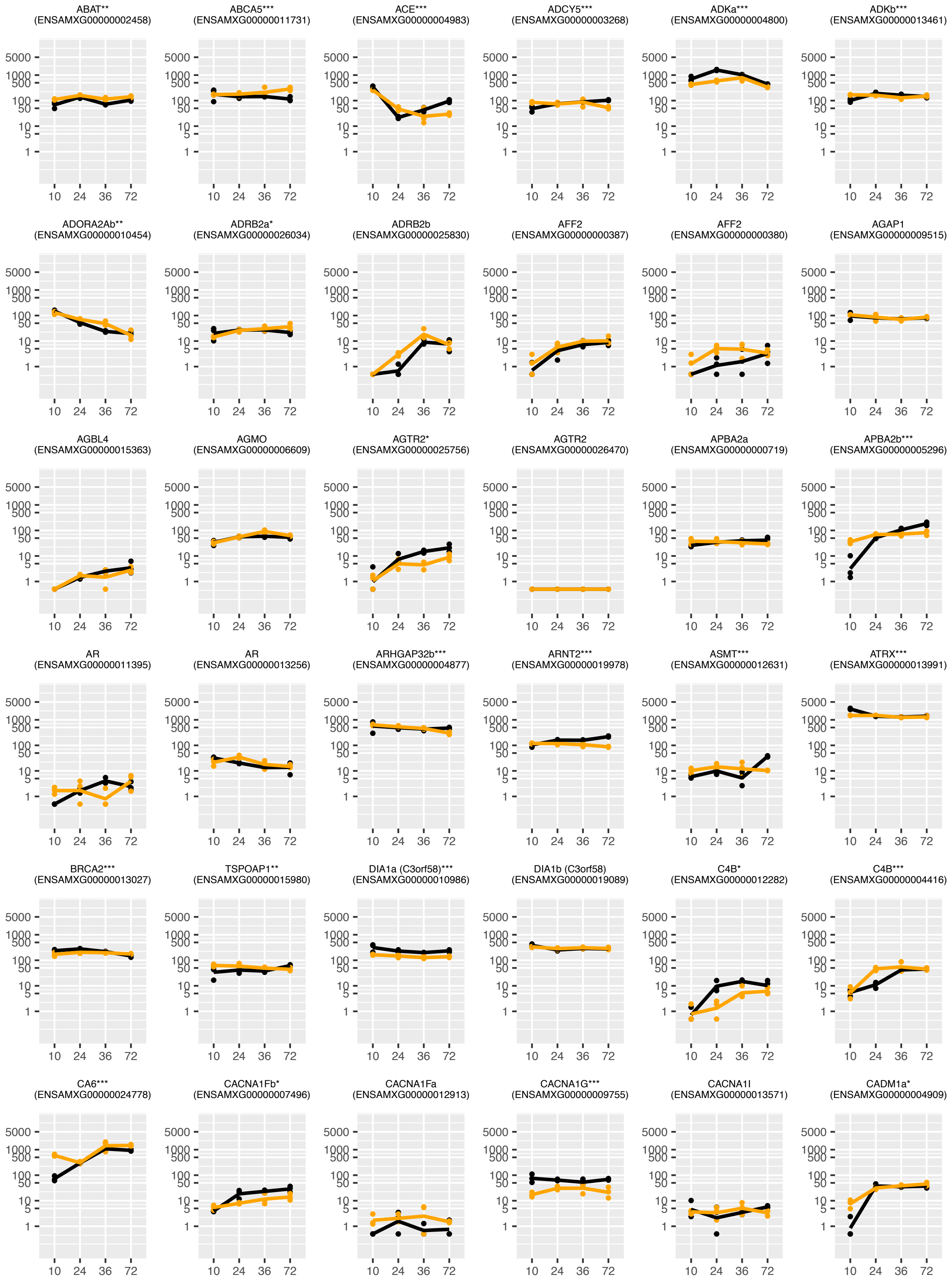

SFARI Gene Category 4 (page 2 of 9)

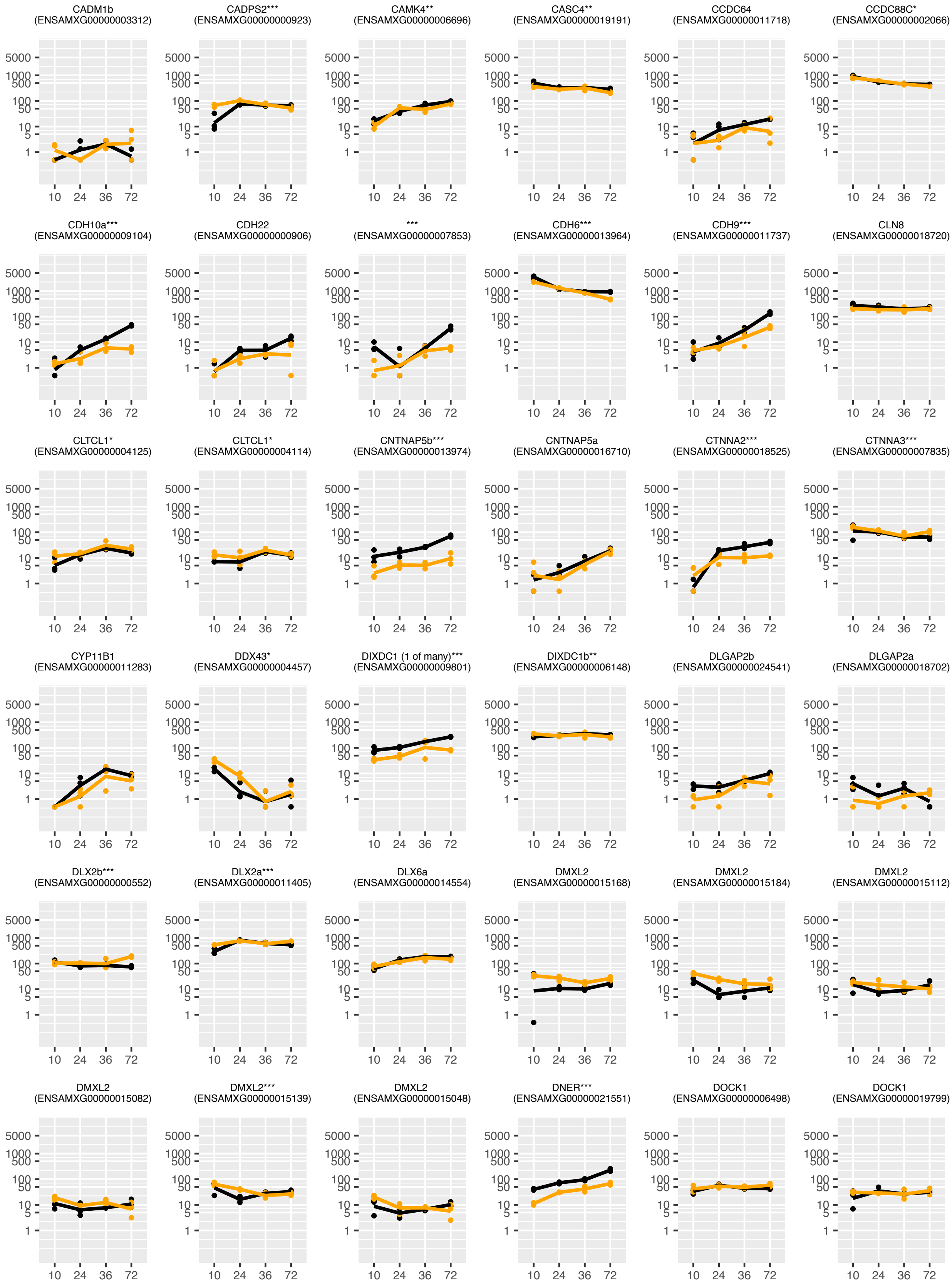

## SFARI Gene Category 4 (page 3 of 9)

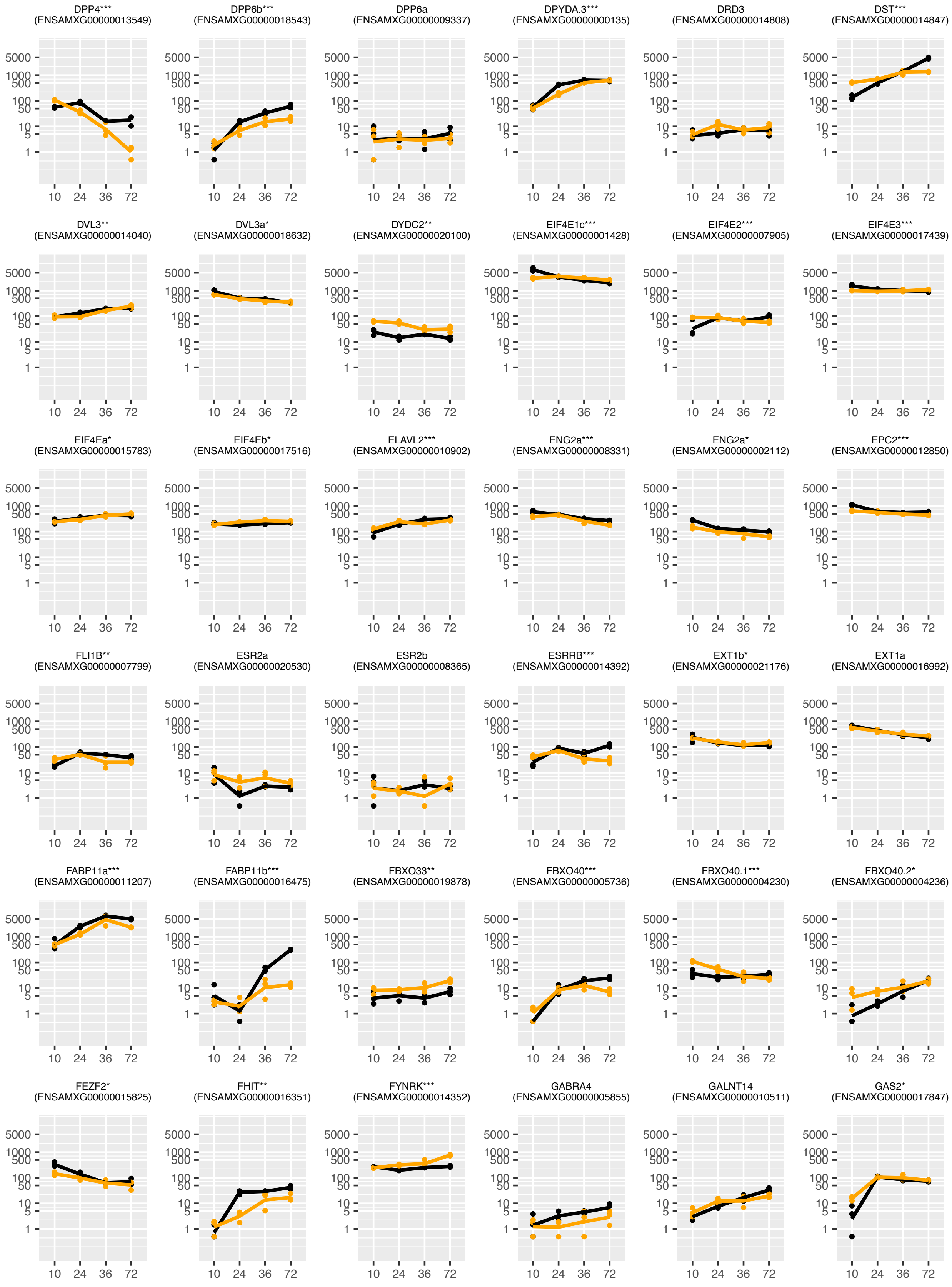

# SFARI Gene Category 4 (page 4 of 9)

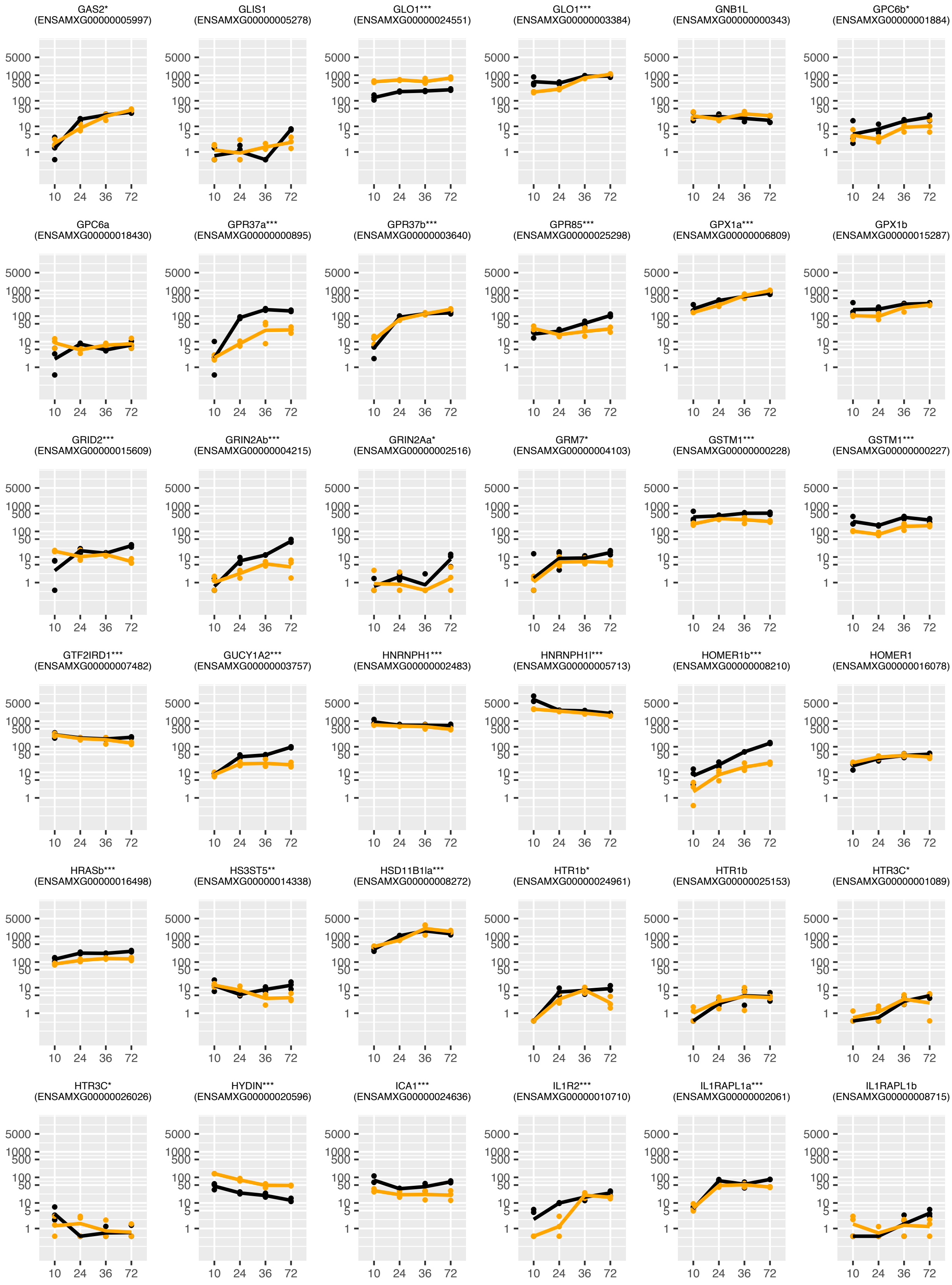

SFARI Gene Category 4 (page 5 of 9)

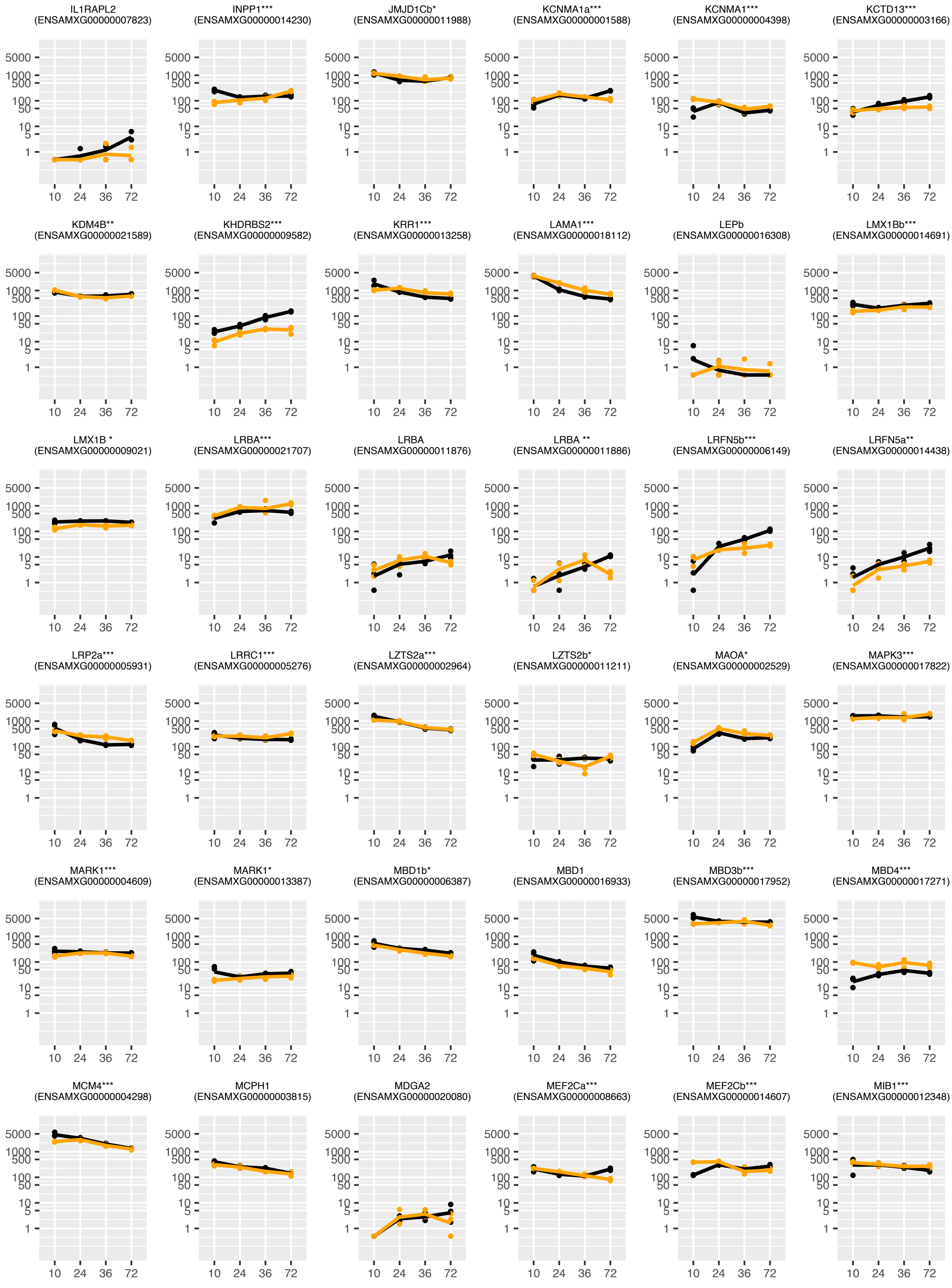

. SFARI Gene Category 4 (page 6 of 9)

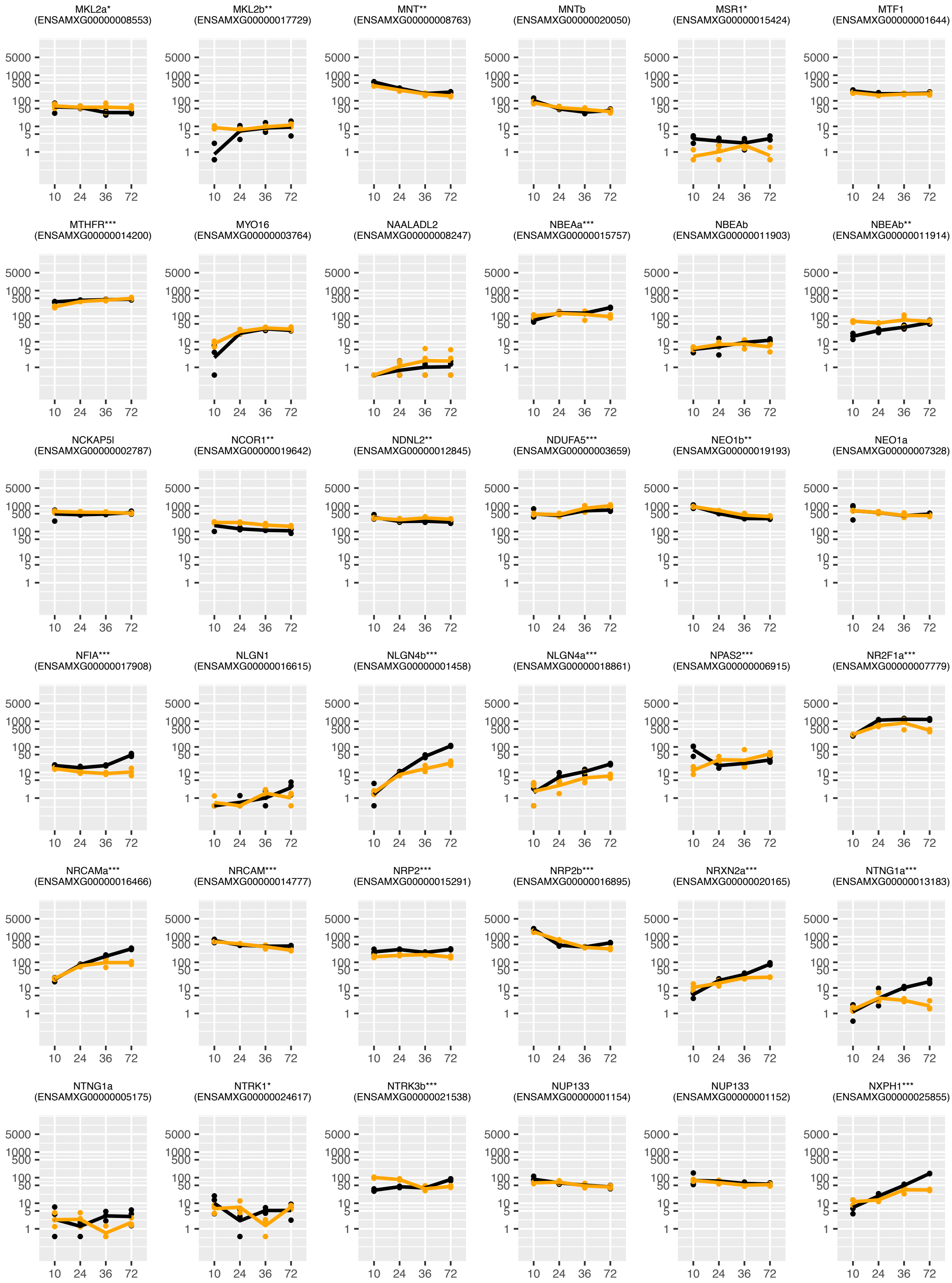

# SFARI Gene Category 4 (page 7 of 9)

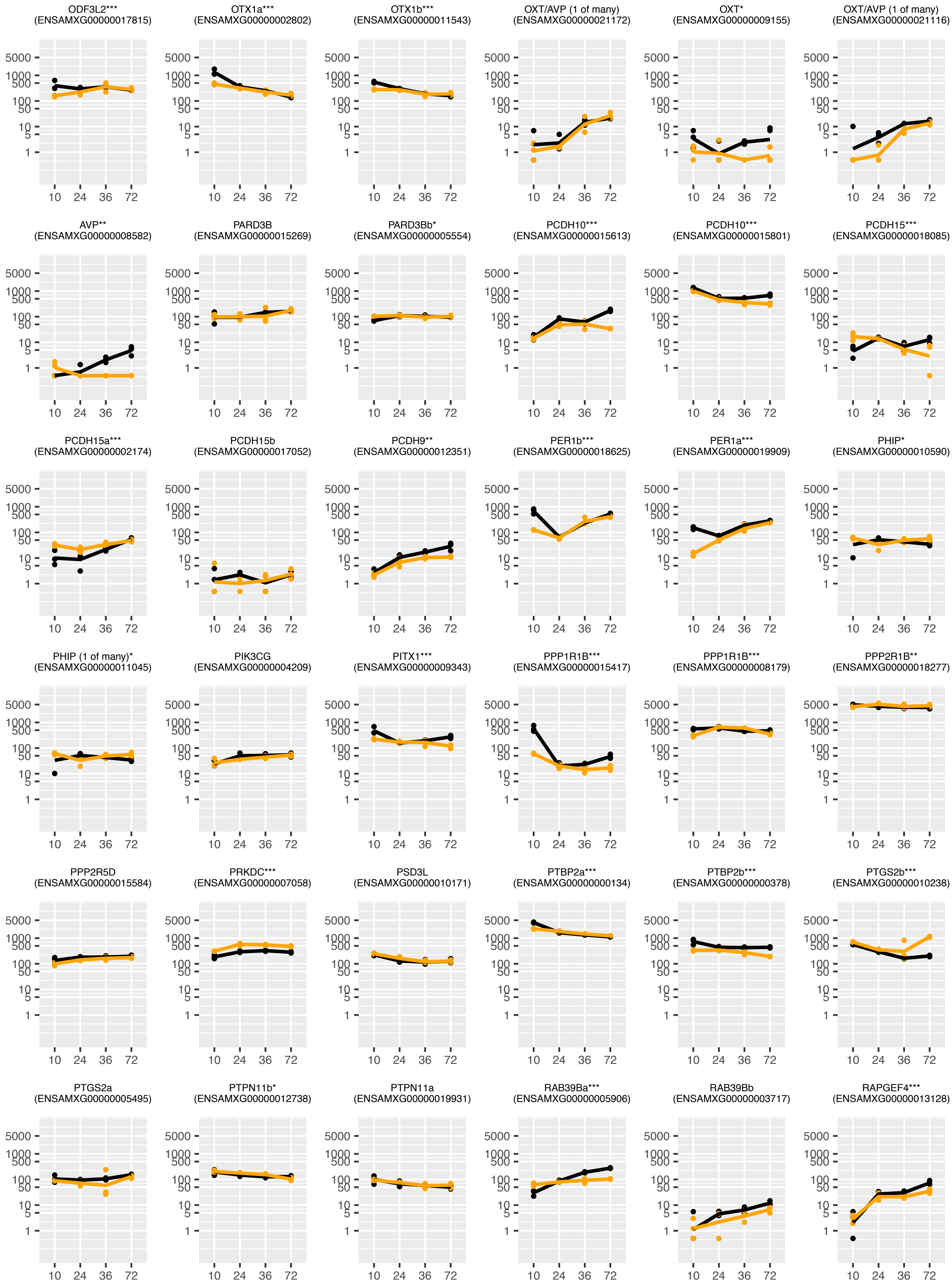

SFARI Gene Category 4 (page 8 of 9)

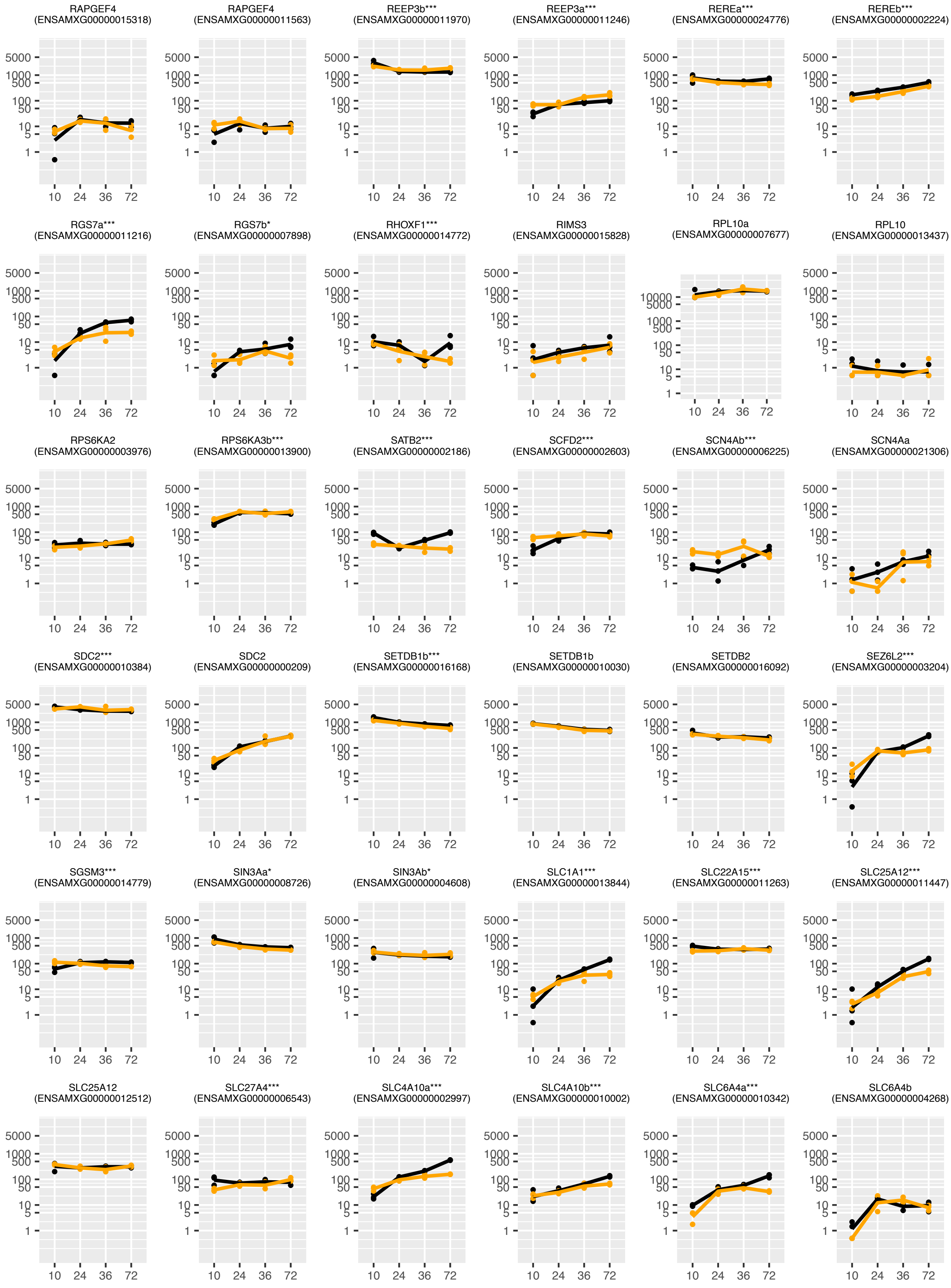

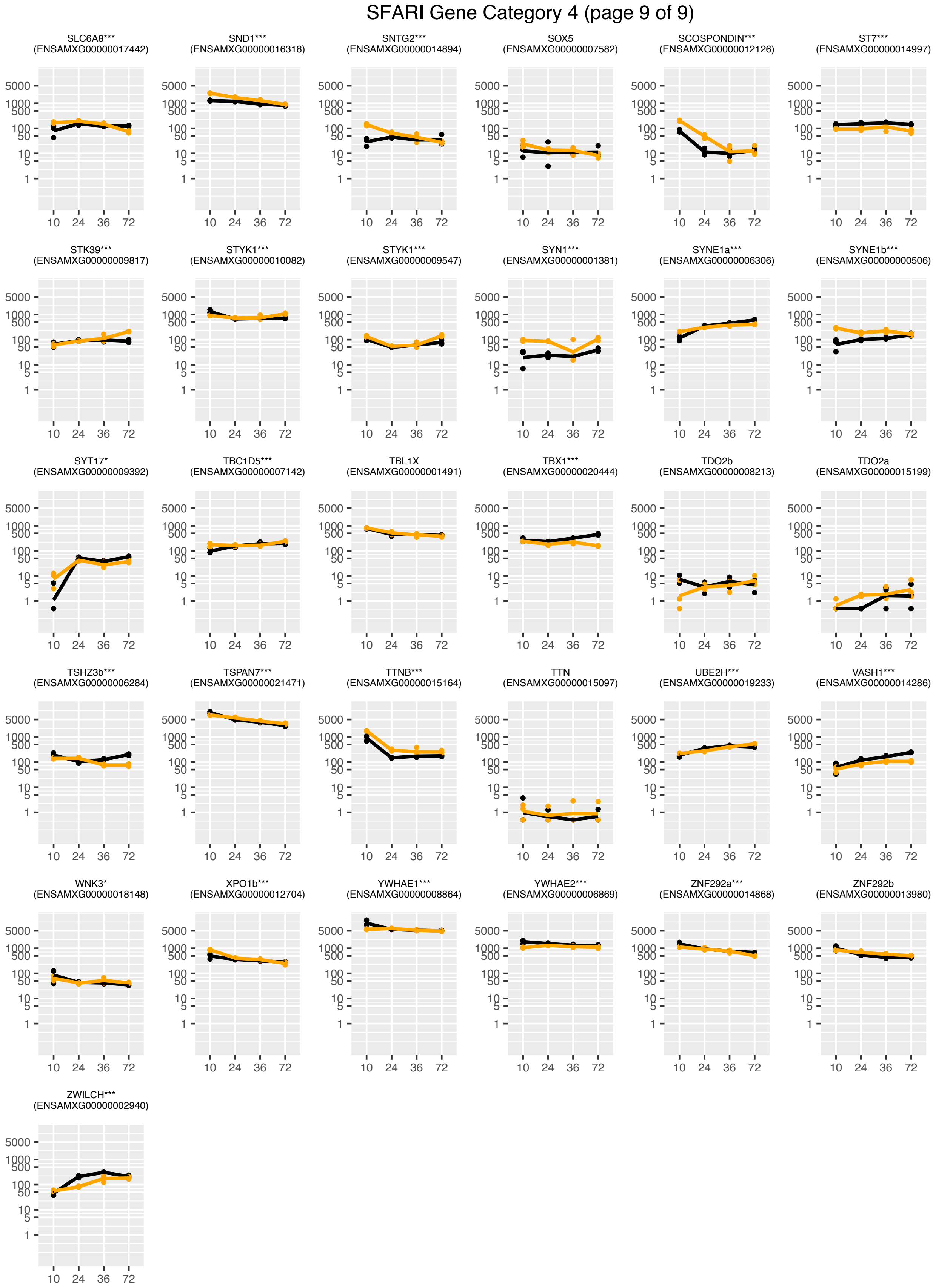
SFARI Gene Category 4 (page 9 of 9)
SLC6A8*** (ENSAMXG00000017442)
SND1*** (ENSAMXG00000016318)
SNTG2*** (ENSAMXG00000014894)
SOX5 (ENSAMXG00000007582)
SCOSPONDIN*** (ENSAMXG00000012126)
ST7*** (ENSAMXG00000014997)
STK39*** (ENSAMXG00000009817)
STYK1*** (ENSAMXG00000010082)
STYK1*** (ENSAMXG00000009547)
SYN1*** (ENSAMXG00000001381)
SYNE1a*** (ENSAMXG00000006306)
SYNE1b*** (ENSAMXG00000000506)
SYT17* (ENSAMXG00000009392)
TBC1D5*** (ENSAMXG00000007142)
TBL1X (ENSAMXG00000001491)
TBX1*** (ENSAMXG00000020444)
TDO2b (ENSAMXG00000008213)
TDO2a (ENSAMXG00000015199)
TSHZ3b*** (ENSAMXG00000006284)
TSPAN7*** (ENSAMXG00000021471)
TTNB*** (ENSAMXG00000015164)
TTN (ENSAMXG00000015097)
UBE2H*** (ENSAMXG00000019233)
VASH1*** (ENSAMXG00000014286)
WNK3* (ENSAMXG00000018148)
XPO1b*** (ENSAMXG00000012704)
YWHAE1*** (ENSAMXG00000008864)
YWHAE2*** (ENSAMXG00000006869)
ZNF292a*** (ENSAMXG00000014868)
ZNF292b (ENSAMXG00000013980)
ZWILCH*** (ENSAMXG00000002940)
5000
1000
500
100
50
10
5
1
10 24 36 72

**Additional file 2.4—associated with Table 1. Many of the ASD-risk genes listed in SFARI Gene Category 4 had significantly different expression in surface fish and cavefish at the stages of 10, 24, 36 and 72 hours post fertilization (hpf).**
A set of 319 (all paralogs of 219 out of 244 orthologs in Category 4) *A. mexicanus* genes that are orthologs of human SFARI Gene Category 4 is shown (https://gene.sfari.org/autdb/GS_Home.do). Y-axis: normalized count of the number of sequence reads (Fragments Per Kilobase Million; FPKM). X-axis: hours post fertilization. Yellow dots and line represent the gene expression of cavefish, and the black dots and line represent that of surface fish. Each dot represents one of 3 replicates in each data point (morph and age). *: $P < 0.05$, **: $P < 0.01$, ***: $P < 0.001$ in the lower P-value of between the age × morph interaction and the expression difference at 72 hpf after applying Benjamini-Hochberg correction [1]. See also Additional file 1.

. SFARI Gene Category S (Syndromic) (page 1 of 4)

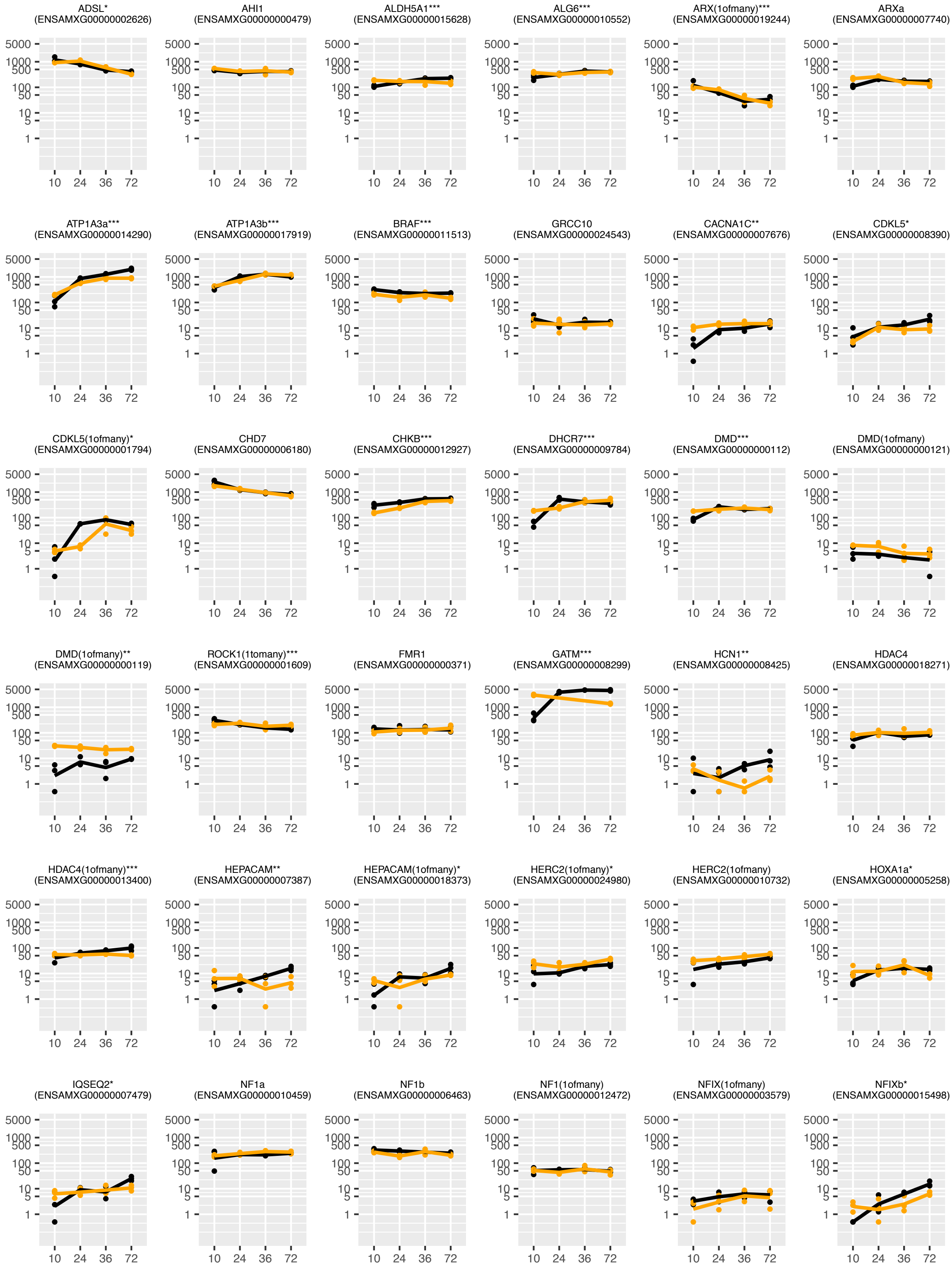

# SFARI Gene Category S (Syndromic) (page 2 of 4)

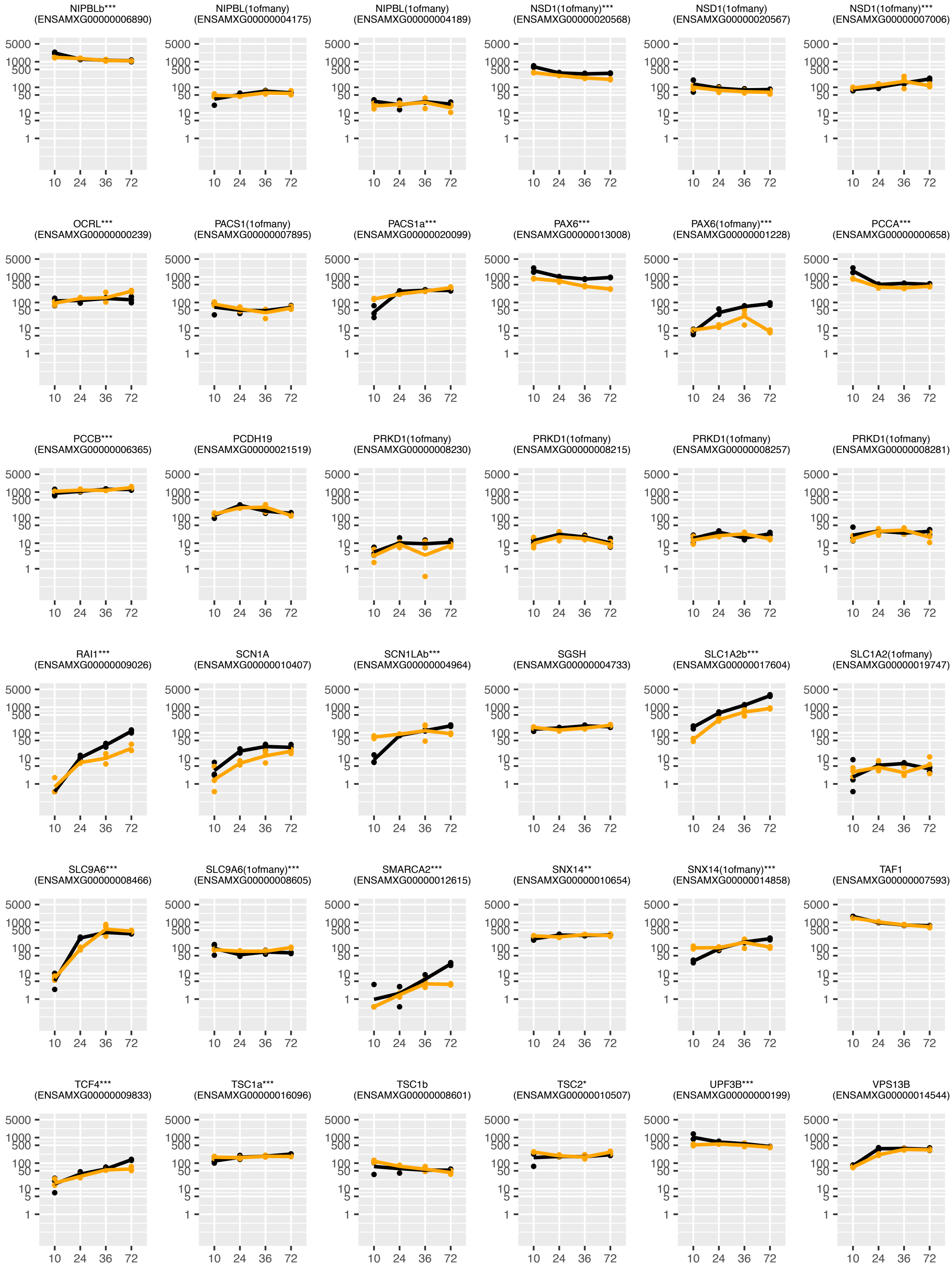

# SFARI Gene Category S (Syndromic) (page 3 of 4)

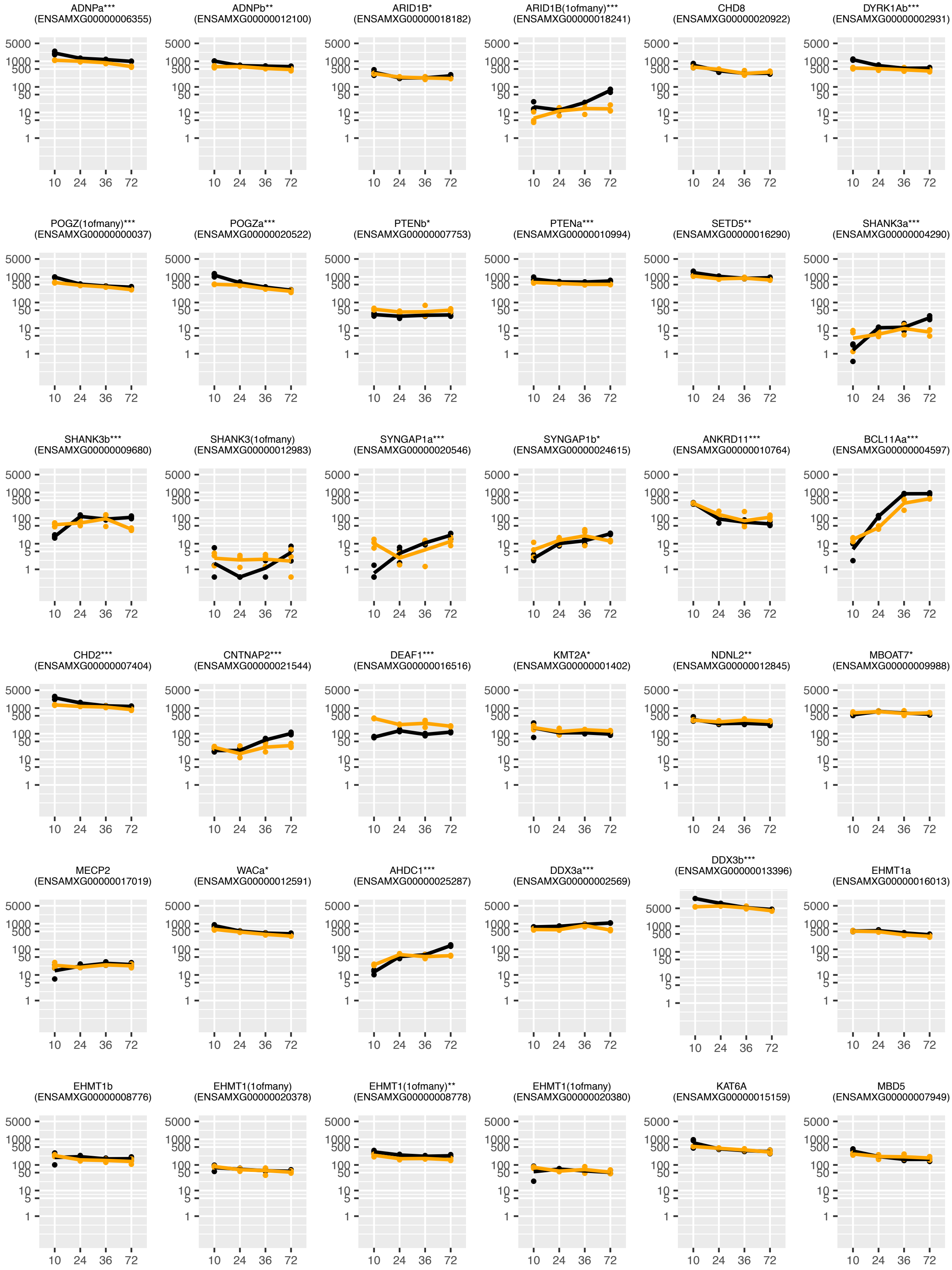

SFARI Gene Category S (Syndromic) (page 4 of 4)

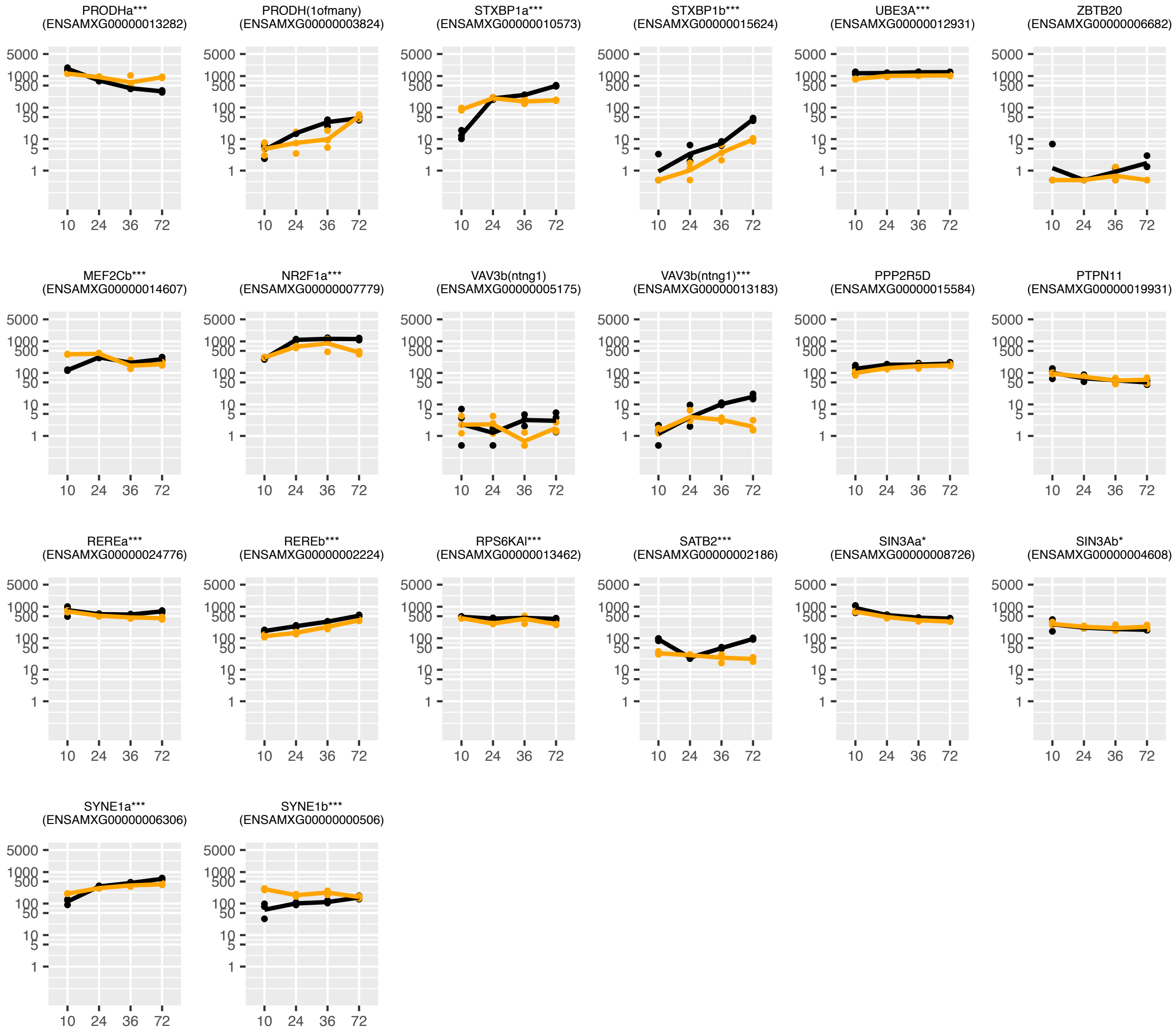


**Additional file 2.5—associated with Table 1. Many of the ASD-risk genes listed in SFARI Gene Category S (Syndromic) had significantly different expression in surface fish and cavefish at the stages of 10, 24, 36 and 72 hours post fertilization (hpf).**

A set of 128 (all paralogs of 85 out of 89 orthologs in Category S including genes which are also listed in Category 1-4. See also Additional file 1) *A. mexicanus* genes that are orthologs of human SFARI Gene Category S (Syndromic) is shown (https://gene.sfari.org/autdb/GS_Home.do). Y-axis: normalized count of the number of sequence reads (Fragments Per Kilobase Million; FPKM). X-axis: hours post fertilization. Yellow dots and line represent the gene expression of cavefish, and the black dots and line represent that of surface fish. Each dot represents one of 3 replicates in each data point (morph and age). *: $P < 0.05$, **: $P < 0.01$, ***: $P < 0.001$ in the lower P-value of between the age × morph interaction and the expression difference at 72 hpf after applying Benjamini-Hochberg correction[1]. See also Additional file 1.

**References:**

1. Love MI, Anders S, Kim V, Huber W, Love MI, Anders S, Kim V, Huber W: **RNA-Seq workflow: gene-level exploratory analysis and differential expression**. *F1000Research* 2016, **4**:1070.
