# Supplementary material for: The evolution of a series of behavioral traits is associated with autism-risk genes in cavefish
Source: BMC Evol Biol. 2018 Jun 18;18:89. doi: 10.1186/s12862-018-1199-9 (PMC6004695; doi:10.1186/s12862-018-1199-9)
Supplement: Supplementary file 3 — Little enrichment of the expression shifts between surface fish and cavefish in SCZ-risk genes listed in SZgene.org or szdb.org. (PDF 235 kb) [file 12862_2018_1199_MOESM3_ESM.pdf]

Additional file 3: S1 . Cavefish and surface fish exhibited differential expression of some of SCZ risk genes.

TOP result at SZgene.org (SCZ) **Genes = 44; NA = 6**

	Symbol	Ensembl id (release 86)	p-value Age-Population interaction	Log2 at 72h	p-value at 72h	Description
1	PDE4Bb	ENSAMXG00000018769	1.48E-15***	1.87	3.48E-68***	phosphodiesterase 4B, cAMP-specific b
1	PDE4Ba	ENSAMXG00000010617	0.15602	0.89	1.08E-19***	phosphodiesterase 4B, cAMP-specific a
2	COMTD1	ENSAMXG00000006675	2.74E-16***	1.05	1.11E-46***	catechol-O-methyltransferase domain containing 1
2	COMTD1	ENSAMXG00000001561	4.31E-05***	-2.28	4.50E-15***	Paralog
3	RGS4	ENSAMXG00000011539	7.35E-07***	1.08	2.74E-39***	regulator of G-protein signaling 4
4	AKT1	ENSAMXG00000009230	1.81E-05***	-1.39	1.09E-22***	AKT serine/threonine kinase 1
4	AKT1	ENSAMXG00000012355	0.31115	-0.21	0.1762	Paralog
5	APOEb	ENSAMXG00000001799	8.55E-14***	0.68	8.07E-14***	apolipoprotein Eb
5	APOEa	ENSAMXG00000009609	0.31422	-0.30	0.5365	apolipoprotein Ea
6	PPP3CCb	ENSAMXG00000020487	0.00787**	0.70	1.19E-12***	protein phosphatase 3, catalytic subunit, gamma isozyme, b
6	PPP3CCa	ENSAMXG00000014038	0.59752	-0.01	0.9801	protein phosphatase 3, catalytic subunit, gamma isozyme, a
7	PLXNA2	ENSAMXG00000001315	2.43E-07***	-1.55	1.47E-10***	plexin A2
8	RELN	ENSAMXG00000015348	1.24E-05***	-1.80	3.79E-10***	reelin
9	IL10RA	ENSAMXG00000004234	2.47E-08***	1.47	4.22E-09***	interleukin 10 receptor, alpha
9	IL10RB	ENSAMXG00000005730	0.05217	-0.35	0.0143*	interleukin 10 receptor, beta
10	TCF4	ENSAMXG00000009833	0.00232**	-1.11	2.76E-08***	transcription factor 4
11	HIST1H4K	ENSAMXG00000025038	0.00171**	-0.83	4.99E-07***	histone cluster 1, H4k
12	ZNF804A	ENSAMXG00000003327	1.03E-05***	-1.07	3.83E-05***	zinc finger protein 804A
13	ARPP21	ENSAMXG00000020587	1.28E-06***	-1.16	0.0002***	cAMP-regulated phosphoprotein, 21
14	DTNBP1a	ENSAMXG00000017514	0.02441*	-0.40	0.0002***	dystrobrevin binding protein 1a
14	DTNBP1b	ENSAMXG00000011399	0.08967	-0.81	0.0824	dystrobrevin binding protein 1b
15	GABRB2	ENSAMXG00000018946	0.00608**	-1.46	0.0005***	gamma-aminobutyric acid (GABA) A receptor, beta 2
15	GABRB2	ENSAMXG00000009424	0.76735	NS	NS	Paralog
16	GRIK3	ENSAMXG00000001749	0.00033***	-1.15	0.0011**	glutamate ionotropic receptor kainate type subunit 3
17	DRD4b	ENSAMXG00000007190	0.00303**	-1.65	0.0073**	dopamine receptor D4b
17	DRD4a	ENSAMXG00000016558	0.07228	-0.21	0.6836	dopamine receptor D4a
18	GRIN2B	ENSAMXG00000007861	0.28463	-1.36	0.0169*	glutamate receptor, ionotropic, N-methyl D-aspartate 2B
18	GRIN2B	ENSAMXG00000007825	0.24203	-1.12	0.0574	Paralog
19	SRRD	ENSAMXG00000006117	0.33004	0.46	0.0243*	SRR1 domain containing
20	DRD2b	ENSAMXG00000007381	0.63264	0.79	0.0339*	dopamine receptor D2b
20	DRD2a	ENSAMXG00000018359	0.00452**	-0.37	0.6117	dopamine receptor D2a
21	NRG1	ENSAMXG00000010103	2.45E-07***	0.38	0.0345*	neuregulin 1
22	DAO.2	ENSAMXG00000013702	0.30373	0.95	0.0439*	D-amino-acid oxidase, tandem duplicate 2
23	SLC18A1	ENSAMXG00000019601	1.70E-12***	0.20	0.6847	solute carrier family 18 member A1
24	COMTA	ENSAMXG00000016773	1.78E-08***	-0.12	0.3453	catechol-O-methyltransferase a
25	NRGNA	ENSAMXG00000007384	4.13E-08***	0.35	0.1756	neurogranin (protein kinase C substrate, RC3) a
26	MTHFR	ENSAMXG00000014200	4.45E-06***	0.15	0.2218	methylenetetrahydrofolate reductase (NAD(P)H)
27	MDGA1	ENSAMXG00000003278	0.00023***	-0.60	0.2873	MAM domain containing glycosylphosphatidylinositol anchor 1
28	RPGRIP1L	ENSAMXG00000017705	0.00348**	0.19	0.2581	RPGRIP1-like
29	OPCML	ENSAMXG00000004329	0.01697*	-0.85	0.0787	opioid binding protein/cell adhesion molecule-like
30	GRIN2Bb	ENSAMXG00000018685	0.13926	-0.99	0.1440	glutamate receptor, ionotropic, N-methyl D-aspartate 2B
30	GRIN2Bb	ENSAMXG00000018677	0.52103	-0.68	0.3288	Paralog
31	TPH1a	ENSAMXG00000018104	0.37913	-0.60	0.0761	tryptophan hydroxylase 1 (tryptophan 5-monooxygenase) a
31	TPH1b	ENSAMXG00000006097	NA	NS	NS	tryptophan hydroxylase 1b
32	HP	ENSAMXG00000007069	0.47585	-1.16	0.0814	haptoglobin
33	HTR2A	ENSAMXG00000012904	0.42769	0.79	0.2168	5-hydroxytryptamine (serotonin) receptor 2A
33	HTR2A	ENSAMXG00000004978	0.97471	-0.11	NS	5-hydroxytryptamine receptor 2A
34	IL10	ENSAMXG00000007244	0.75621	0.40	0.4648	interleukin 10
35	RPP21	ENSAMXG00000021554	0.31111	0.16	0.4672	ribonuclease P 21 subunit
36	CCKAR	ENSAMXG00000010814	0.19380	-0.23	0.6901	cholecystokinin A receptor
37	PRSS16	ENSAMXG00000014906	0.55529	0.15	0.8476	protease, serine, 16
38	AHI1	ENSAMXG00000000479	0.71473	-0.01	0.9389	Abelson helper integration site 1
39	DAOA	NA	NA	NA	NA	D-amino acid oxidase activator
40	DISC1	NA	NA	NA	NA	disrupted in schizophrenia 1
41	HIST1H2BJ	NA	NA	NA	NA	histone cluster 1, H2bj
42	IL2B	NA	NA	NA	NA	Interleukin 2
43	NOTCH4	NA	NA	NA	NA	notch 4
44	PGBD1	NA	NA	NA	NA	piggyBac transposable element derived 1

P-values are calculated for the age-population interaction (surface fish vs. Pachón cavefish populations at 10 hours post fertilization (hpf), 24 hpf, 32 hpf, and 72 hpf) from a two-way ANOVA. We also report the expression difference between surface fish and Pachón cavefish at 72 hpf as $\log_2$ and P-values adjusted by the Benjamini-Hochberg method[1, 2]. *: P < 0.05, **: P < 0.01, ***: P < 0.001. In SZ genes, 50.9 % had a significant age × morph interaction and 49.0% had a significant main effect of morph type at 72 hpf, which were not significantly different from the random set. See Additional file 3.2 too.

Additional file 3: S2. Little enrichment of the expression shifts between surface fish and cavefish in SCZ-risk genes listed in szdb.org[3].

Human SCZ-risk genes (szdb.org)		Cavefish genes				
Risk Category	# of Listed Genes in szdb.org	% (#) human ASD-risk genes with cavefish orthologs	% (#) of orthologs that show significant age × morph interaction†	% (#) of orthologs that show significant expression difference between morphs at 72 hpf†	% (#) of all paralogs that show significant age × morph interaction†	% (#) of all paralogs that show significant expression difference between morphs at 72 hpf†
Score 4	3	**66.7 %** (2)	**100.0 %** (2)	**100.0 %** (2)	**40.0 %** (P>0.99) (<0.1 percentile) (2 of 5)	**40.0 %** (<0.1 percentile) (2 of 5)
Score 3	26	**80.8 %** (21)	**57.1 %** (12)	**66.7 %** (14)	**51.7 %** (**95.8 percentile**) (15 of 29)	**55.2 %** (**99.7 percentile**) (16 of 29)
Score 2	275	**79.6 %** (219)	**46.6 %** (102)	**61.6 %** (135)	**39.8 %** (<0.1 percentile) (130 of 327)	**51.1 %** (82.2 percentile) (167 of 327)
**Total**	304 (score 4-2)	**79.6 %** (242)	**62.1 %** (283)	**66.9 %** (305)	**40.7 %** (147 of 361)	**51.2 %** (185 of 361)
Bootstrapping score: mean ± 95% confidence interval					**48.0 ± 4.2 %**	**49.0 ± 4.4 %**

Score 4 genes show the most evidences as SCZ risk genes and Score 1 genes are the least [3]. The 2,402 Score 1 genes were not analyzed in this study. † $P < 0.05$ after Benjamini-Hochberg adjustment. Percentiles in the tables are from 9,999-bootstrapped values. SF: surface fish. CF: Pachón cavefish. hpf: hours post fertilization. See also Table 1 and Additional file 3.1.

References:

1. Love MI, Anders S, Kim V, Huber W, Love MI, Anders S, Kim V, Huber W: **RNA-Seq workflow: gene-level exploratory analysis and differential expression**. *F1000Research* 2016, **4**:1070.
2. Benjamini Y, Hochberg Y: **Controlling the False Discovery Rate: A Practical and Powerful Approach to Multiple Testing**. *J R Stat Soc Ser B* 1995, **57**:289–300.
3. Wu Y, Yao Y-G, Luo X-J: **SZDB: A Database for Schizophrenia Genetic Research**. *Schizophr Bull* 2016, **43**:459–471.
