# Supplementary material for: The evolution of a series of behavioral traits is associated with autism-risk genes in cavefish
Source: BMC Evol Biol. 2018 Jun 18;18:89. doi: 10.1186/s12862-018-1199-9 (PMC6004695; doi:10.1186/s12862-018-1199-9)
Supplement: Supplementary file 5 — Example expression patterns of ASD-risk genes in SFARI Gene Category 1. Quantitative RT-PCR showed similarities to ASD patients. (PDF 74 kb) [file 12862_2018_1199_MOESM5_ESM.pdf]

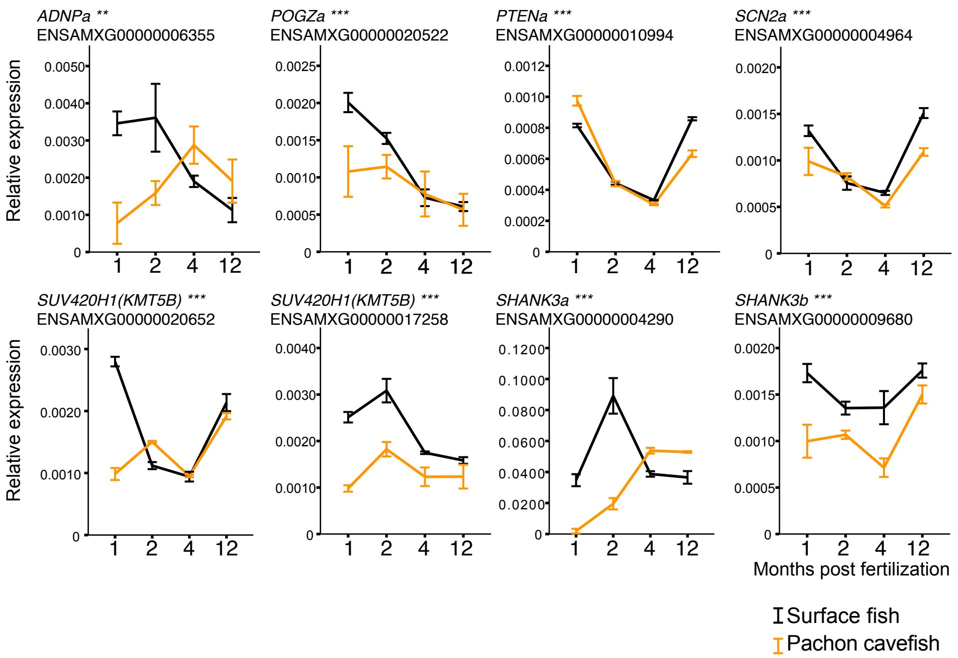

Example expression patterns of ASD-risk genes in SFARI Gene Category 1: Quantitative RT-PCR showed similarities to ASD patients.

mRNA were extracted from brain tissues from both morphs at the age of 1 month, 2 months, 4 months, and 1 year old. Due to the anatomy and physiology of *Astyanax* (also c.f. [1], we consider fish larvae younger than 5 days old to be roughly comparable to a human embryo; fish juvenile 5 days to 4 months old (free swimming with immature gonadal organs) are comparable to the human infant to the juvenile stage; fish sub-adults 4-6 months old can be compared to the humans at puberty; and fish adults 6 months to 1 year old are comparable to young to mature human adults. Each data point represents mean ± s.e.m. of 3 technical replicates using the same cDNA library (see Methods). Black: surface fish, Orange: cavefish. Eight example genes in the SFARI Gene Category 1 showed significantly different profiles of gene expression between surface fish and cavefish (See the table below). Note that the expression of surface fish *shank3a* showed a peak at the juvenile stage (2 months post fertilization), while *shank3a* in cavefish did not exhibit this peak. This peak can also be absent in ASD patients, due to over-methylation which attenuates *SHANK3* expression over development [2, 3]. *: $P < 0.05$, **: $P < 0.01$, ***: $P < 0.001$.

Two-way ANOVA for qRT-PCR results from three technical replicates

		F stats	P-values
adnp	Population	F(1,16) = 4.2	0.056
ENSAMXG00000006355	Age	F(3,16) = 1.7	0.207
	Pop × Age	F(3,16) = 6.9 **	0.003
pogza	Population	F(1,16) = 5.3 *	0.034
ENSAMXG00000020522	Age	F(3,16) = 10.6 ***	<0.001
	Pop × Age	F(3,16) = 2.5	0.099
ptena	Population	F(1,16) = 4.6 *	0.048
ENSAMXG00000010994	Age	F(3,16) = 548.1 ***	<0.001
	Pop × Age	F(3,16) = 48.4 ***	<0.001
scn2a	Population	F(1,16) = 18.3 ***	<0.001
ENSAMXG00000004964	Age	F(3,16) = 47.4 ***	<0.001
	Pop × Age	F(3,16) = 5.1 **	0.011
suv420h1 (kmt5)	Population	F(1,16) = 57.0 ***	<0.001
ENSAMXG00000020652	Age	F(3,16) = 86.1 ***	<0.001
	Pop × Age	F(3,16) = 79.2 ***	<0.001
suv420h1 (kmt5)	Population	F(1,16) = 61.6 ***	<0.001
ENSAMXG00000017258	Age	F(3,16) = 16.6 ***	<0.001
	Pop × Age	F(3,16) = 6.0 **	0.006
shank3a	Population	F(1,16) = 27.2 ***	<0.001
ENSAMXG00000004290	Age	F(3,16) = 21.1 ***	<0.001
	Pop × Age	F(3,16) = 37.0 ***	<0.001
shank3b	Population	F(1,16) = 35.2 ***	<0.001
ENSAMXG00000009680	Age	F(3,16) = 9.7 ***	<0.001
	Pop × Age	F(3,16) = 2.3	0.120
