# Supplementary material for: The evolution of a series of behavioral traits is associated with autism-risk genes in cavefish
Source: BMC Evol Biol. 2018 Jun 18;18:89. doi: 10.1186/s12862-018-1199-9 (PMC6004695; doi:10.1186/s12862-018-1199-9)
Supplement: Supplementary file 6 — DXY and FST statistics and plots for the top outliers whose divergence metrics passed our threshold (i.e. top 5% for FST, top 20% for DXY, and P < 0.05 for hapFLK). (PDF 139 kb) [file 12862_2018_1199_MOESM6_ESM.pdf]

Additional file 6.1. Means (± standard error of means) and numbers of genes for F_{ST} and D_{XY} measures between passed and non-passed either of our thresholds (top 5% of F_{ST}, top 20% of D_{XY})

	ASD genes passed the threshold	non-ASD genes passed the threshold	ASD genes did not pass the threshold	non-ASD genes did not pass the threshold
F_{ST}	0.634 ± 0.017	0.667 ± 0.003	0.204± 0.005	0.230 ± 0.001
N	19§	918	621§	20002
D_{XY}	0.005 ± <0.001	0.010 ± <0.001	0.002 ± <0.001	0.002 ± <0.001
N	51§	4228	589§	16745

§ Note that total ASD genes for F_{ST} and D_{XY} are lower than the total for orthologs and paralogs as we removed any genes that had missing values for F_{ST} and D_{XY} and removed any individuals with cave Pi gene rank < 500 (see Additional file 6.2, Additional file 7 and *Gene enrichment analysis* in Materials and Methods).

Additional file 6.2. Comparisons between ASD-risk genes and non-ASD-risk genes, which are both passed or did not passed either of our cut-off thresholds (top 5% for F_{ST}, top 20% for D_{XY} or P < 0.05 for hapFLK) (see also Additional file 6.1 and Additional file 7).

	**Kruskal-Wallis comparisons**	**Chi-square X^2**	**Degrees of freedom**	**P-value**
F_{ST}	ASD genes vs. non-ASD genes of both passed the threshold	1.727	1	0.189
	ASD genes vs. non-ASD genes of both did not pass the threshold	4.781	1	0.029
D_{XY}	ASD genes vs. non-ASD genes of both passed the threshold	29.285	1	<0.001
	ASD genes vs. non-ASD genes of both did not pass the threshold	0.046	1	0.830

Reported are the results based on the Kruskal-wallis test (*kruskal.test* in R). The numbers reported are based on the means for F_{ST} and D_{XY} (see Additional file 6.1 and Additional file 7).

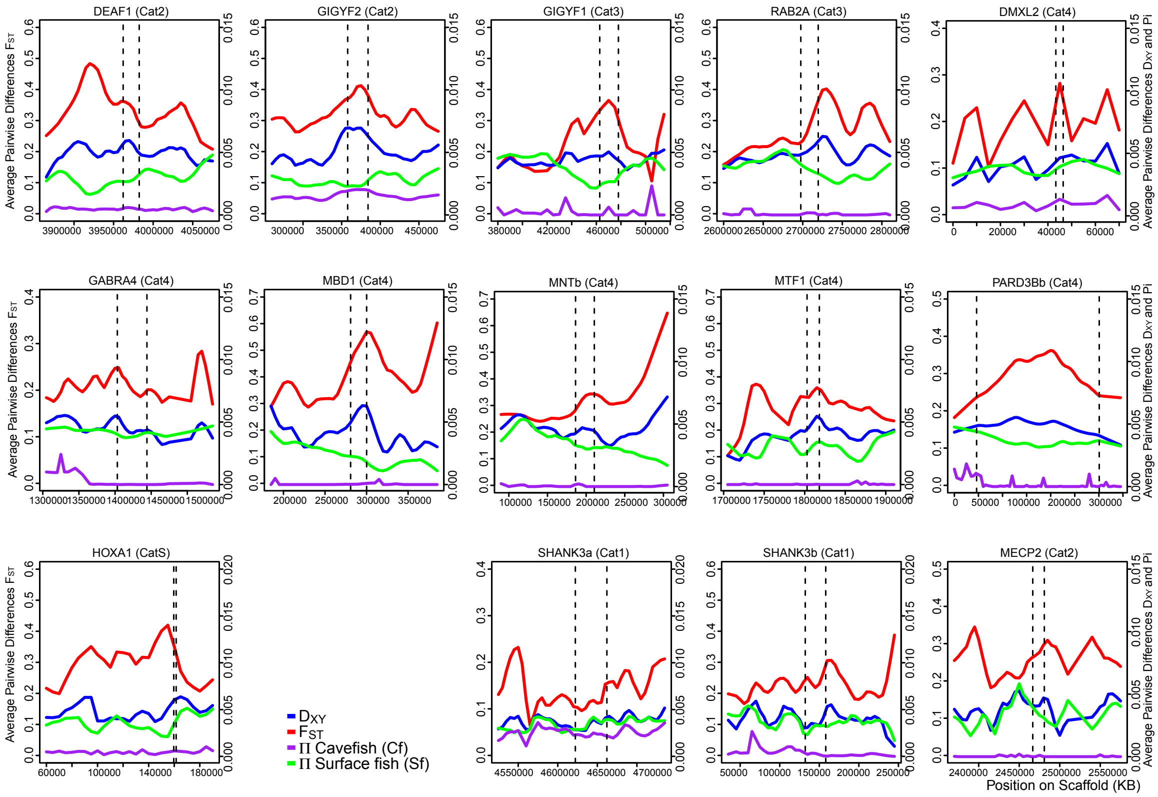

Additional file 6.3. F_{ST}, D_{XY} and Pi plots for the top outliers whose either of two of three divergence metrics passed our threshold (i.e. top 5 % for F_{ST}, top 20 % for D_{XY}, and P < 0.05 for hapFLK).

Many of top outliers show higher divergence scores of F_{ST} (red lines) or D_{XY} (blue lines) around gene interval (between black dotted lines). For reference, two examples of non-outliers but classic ASD-associated genes (*shank3* paralogs and *mecp2*) are included at the right bottom. X-axis indicates positions (Kilo bases) on the genomic scaffold of *Astyanax* genome sequence (AstMex102, www.Ensembl.org). The left Y-axis corresponds to measures of F_{ST}, whereas the right Y-axis is associated with D_{XY} and cave and surface Pi. See also Additional file 7. Cat: Category in SFARI genes[1, 2]

References:

1. Basu SN, Kollu R, Banerjee-Basu S: **AutDB: a gene reference resource for autism research**. *Nucleic Acids Res* 2009, **37**(Database):D832–D836.
2. Abrahams BS, Arking DE, Campbell DB, Mefford HC, Morrow EM, Weiss LA, Menashe I, Wadkins T, Banerjee-Basu S, Packer A: **SFARI Gene 2.0: a community-driven knowledgebase for the autism spectrum disorders (ASDs).** *Mol Autism* 2013, **4**:36.
