# Supplementary material for: The evolution of a series of behavioral traits is associated with autism-risk genes in cavefish
Source: BMC Evol Biol. 2018 Jun 18;18:89. doi: 10.1186/s12862-018-1199-9 (PMC6004695; doi:10.1186/s12862-018-1199-9)
Supplement: Supplementary file 8 — Ingenuity Pathway Analysis Comparison Analysis revealed enriched pathway categories in diversified SFARI genes in cavefish. (PDF 8084 kb) [file 12862_2018_1199_MOESM8_ESM.pdf]

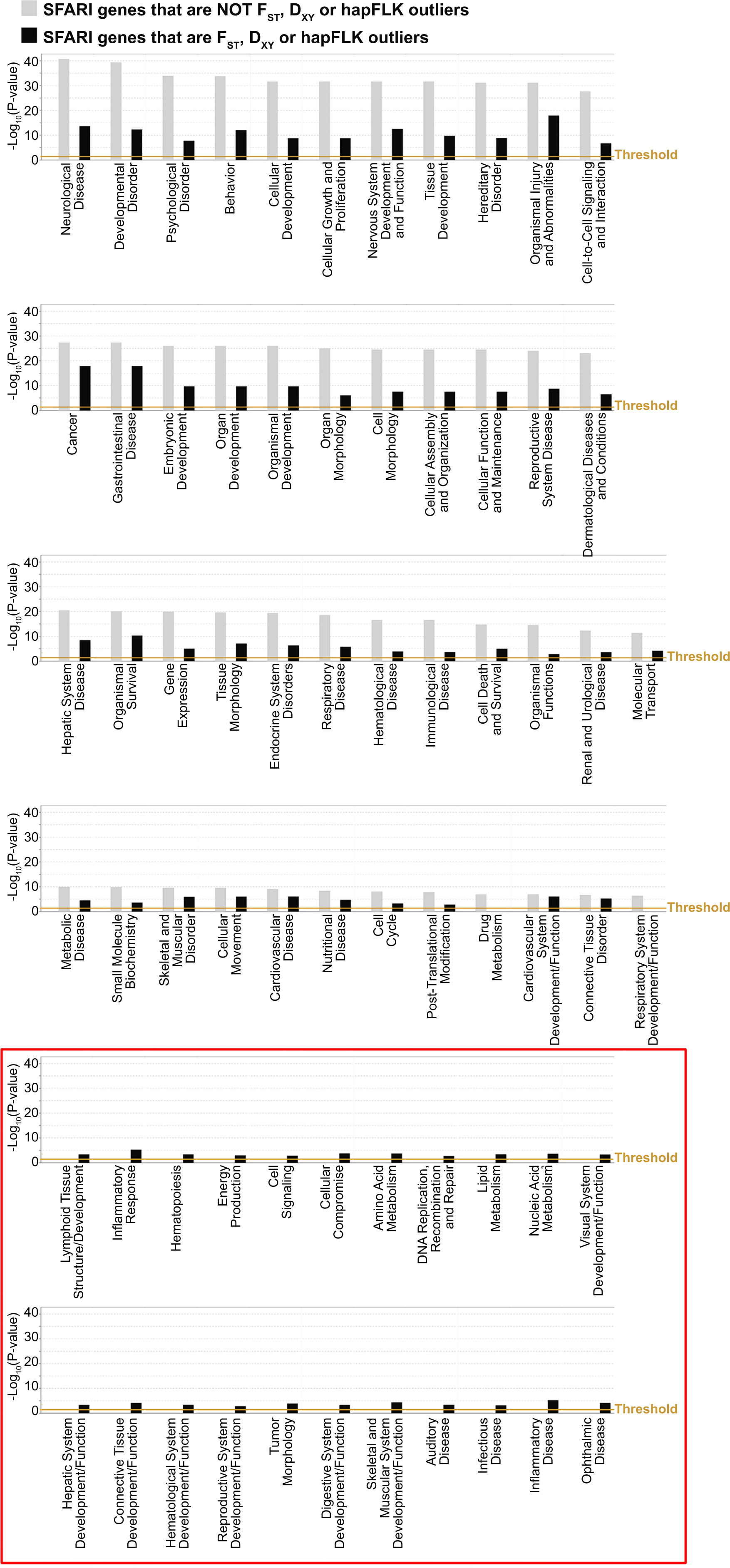

Additional file 8. Ingenuity Pathway Analysis Comparison Analysis [47] revealed enriched pathway categories in diversified ASD-risk genes in cavefish.
In comparison with ASD-risk genes (SFARI genes) which did not pass either of our thresholds (top 5 % for F_{ST}, top 20 % for D_{XY} or $P < 0.05$ for hapFLK), ASD-risk genes passed our threshold highlighted the unique pathway categories including digestive system development and function, inflammatory diseases, lipid metabolism, and energy metabolism (enclosed with a red rectangle). The enriched pathways that passed the significant threshold for this analysis are shown (above the yellow line).
