# Supplementary material for: The evolution of a series of behavioral traits is associated with autism-risk genes in cavefish
Source: BMC Evol Biol. 2018 Jun 18;18:89. doi: 10.1186/s12862-018-1199-9 (PMC6004695; doi:10.1186/s12862-018-1199-9)
Supplement: Supplementary file 10 — Human drugs for psychiatric disease mitigated cavefish-type symptoms in a dose-dependent manner. (PDF 93 kb) [file 12862_2018_1199_MOESM10_ESM.pdf]

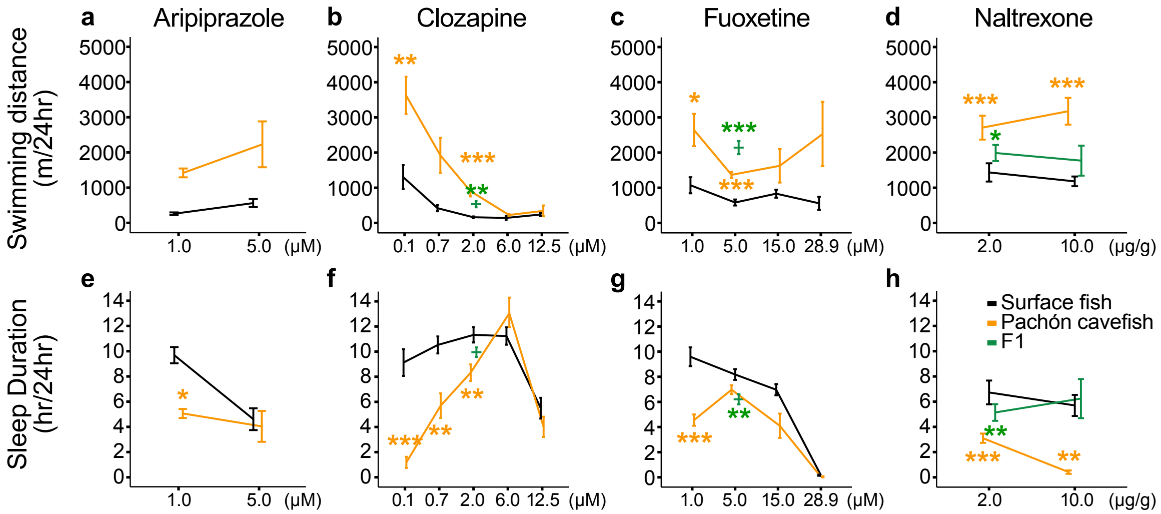

Additional file 10.1. Human drugs for psychiatric disease mitigated cavefish-type symptoms in a dose-dependent manner.

(a-d) Swimming distance per 24 hours assayed under the different drug concentrations (mean ± s.e.m.: m). **(e-h)** Sleep duration per 24 hours assayed under the different drug concentrations (hours). Note that in the control (no drugs), surface fish, Pachón cavefish and the F_1 hybrid slept 6.4 ± 0.4 hours, 1.8 ± 0.2 hours and 4.2 ± 0.4 hours (mean ± s.e.m.), respectively (not shown here). The same individuals were used in the swimming distance assay (a-d). Note that in the control (no drugs), surface fish, cavefish and their F_1 hybrid swam 1,249 ± 109 m, 3,495 ± 226 m and 3,150 ± 281 m (mean ± s.e.m.), respectively (not shown here). Drug concentrations were determined by referring to previous experiments[1–3]. N = 88, 85 and 43 for surface fish, Pachón cavefish and F_1 hybrid in total, respectively. All statistical scores are available in Additional file 9.2.

(a, e) Aripiprazole dose response in swimming distance and sleep duration. Pachón cavefish significantly reduced swimming distance and recovered sleep duration with 1 μM of the aripiprazole treatment. Fish survival was compromised at 5 μM.

(b, f) Clozapine dose response in swimming distance and sleep duration. Cavefish reduced hyperactivity and recovered sleep duration at higher doses. The response of F_1 hybrid was only observed at 2 μM (see Figure 3, too). Fish survival was compromised at 12.5 μM.

(c, g) Fluoxetine hydrochloride dose response in swimming distance and sleep duration. Cavefish reduced hyperactivity and recovered sleep with a peak at 5 μM. The drug response was measured only at this peak point in F_1 hybrids (see Figure 3, too). Fish survival was compromised at 28.9 μM.

(d, h) Naltrexone hydrochloride dose response in swimming distance and sleep duration. Both cavefish and surface fish didn't change their behavior in 2 or 10 μg/body weight g.

Black line: surface fish, orange line: cavefish, and green line: F_1 hybrid. For some drugs, only a single concentration was tested for F_1 hybrids.

*: $P < 0.05$, **: $P < 0.01$, ***: $P < 0.001$ comparing with surface fish at the same drug concentration with Bonferroni correction.

Additional file 10.2. Statistical scores for Additional file 9.1

**Fluoxetine-HCl**		F stats	P-values
Sleep duration	Population	$F(1,72) = 43.5$	<0.001
	Dose	$F(2,72) = 7.3$	0.0
	Pop × Dose	$F(2,72) = 6.4$	0.0
Swimming distance	Population	$F(1,72) = 28.1$	<0.001
	Dose	$F(2,72) = 7.3$	0.0
	Pop × Dose	$F(2,72) = 1.6$	0.2

**N**	1.0	5.0	15.0	(28.9)	(μM)
Surface fish	10	18	10	5	
Pachón cavefish	10	20	10	3	
				(lethal dose)	

**Clozapine**			
Sleep duration	Population	$F(1,77) = 30.2$	<0.001
	Dose	$F(3,77) = 21.2$	<0.001
	Pop × Dose	$F(3,77) = 8.1$	<0.001
Swimming distance	Population	$F(1,77) = 32.1$	<0.001
	Dose	$F(3,77) = 24.7$	<0.001
	Pop × Dose	$F(3,77) = 5.5$	0.0

**N**	0.1	0.7	2.0	6.0	(12.5)	(μM)
Surface fish	8	10	19	5	5	
Pachón cavefish	10	10	18	5	5	
					(lethal dose)	

**Naltrexone-HCl**			
Sleep duration	Population	$F(1,52) = 25.1$	<0.001
	Dose	$F(1,52) = 4.3$	0.0
	Pop × Dose	$F(1,52) = 0.9$	0.3
Swimming distance	Population	$F(1,52) = 23.1$	<0.001
	Dose	$F(1,52) = 0.2$	0.6
	Pop × Dose	$F(1,52) = 0.7$	0.4

**N**	2.0	10.0	(μg/ body weight g)
Surface fish	20	8	
Pachón cavefish	20	8	

**Aripiprazole**			
Sleep duration	Population	$F(1,45) = 21.1$	<0.001
	Before and After treatment	$F(1,45) = 3.3$	0.1
	Pop × BfAf	$F(1,45) = 7.9$	0.0
Swimming distance	Population	$F(1,45) = 21.5$	<0.001
	Before and After treatment	$F(1,45) = 69.6$	<0.001
	Pop × BfAf	$F(1,45) = 39.8$	<0.001

**N**	1.0	(5.0)	(μM)
Surface fish	23	10	
Pachón cavefish	24	10	
		(lethal dose)	

BfAf: Before and after the drug treatment. Pop: population

References

1. Rihel J, Prober DA, Arvanites A, Lam K, Zimmerman S, Jang S, Haggarty SJ, Kokel D, Rubin LL, Peterson RT, Schier AF: **Zebrafish behavioral profiling links drugs to biological targets and rest/wake regulation.** *Science* 2010, **327**:348–51.
2. Stewart A, Wu N, Cachat J, Hart P, Gaikwad S, Wong K, Utterback E, Gilder T, Kyzar E, Newman A, Carlos D, Chang K, Hook M, Rhymes C, Caffery M, Greenberg M, Zadina J, Kalueff A V.: **Pharmacological modulation of anxiety-like phenotypes in adult zebrafish behavioral models**. *Prog Neuro-Psychopharmacology Biol Psychiatry* 2011, **35**:1421–1431.
3. Stewart AM, Nguyen M, Wong K, Poudel MK, Kalueff A V.: **Developing zebrafish models of autism spectrum disorder (ASD)**. *Prog Neuro-Psychopharmacology Biol Psychiatry* 2014:27–36.
