# Supplementary material for: The evolution of a series of behavioral traits is associated with autism-risk genes in cavefish
Source: BMC Evol Biol. 2018 Jun 18;18:89. doi: 10.1186/s12862-018-1199-9 (PMC6004695; doi:10.1186/s12862-018-1199-9)
Supplement: Supplementary file 11 — Statistical scores for Fig. 2. (PDF 45 kb) [file 12862_2018_1199_MOESM11_ESM.pdf]

Statistical scores for Figure 2.

**Clozapine** (2 μM, repeated two-way ANOVA)		F statistics	P-values
Sleep duration	Population	F(2,62) = 19.1	<0.001
	Before and After treatment	F(1,62) = 213.0	<0.001
	Pop × BfAf	F(2,62) = 2.4	0.097
Swimming distance	Population	F(2,62) = 16.9	<0.001
	Before and After treatment	F(1,62) = 91.5	<0.001
	Pop × BfAf	F(2,62) = 12.7	<0.001
Vibration attraction	Population	F(2,61) = 20.6	<0.001
	Before and After treatment	F(1,61) = 4.3	0.041
	Pop × BfAf	F(2,61) = 17.9	<0.001

**N**	2.0 (μM)
Surface fish	19
Pachón cavefish	18
F1 hybrid	28

**N**	2.0 (μM)
Surface fish	24
Pachón cavefish	21
F1 hybrid	19

**Fluoxetine-HCl** (5 μM, repeated two-way ANOVA)			
Sleep duration	Population	F(2,65) = 37.5	<0.001
	Before and After treatment	F(1,65) = 50.8	<0.001
	Pop × BfAf	F(2,65) = 43.7	<0.001
Swimming distance	Population	F(2,65) = 36.4	<0.001
	Before and After treatment	F(1,65) = 83.6	<0.001
	Pop × BfAf	F(2,65) = 33.9	<0.001
Vibration attraction	Population	F(2,86) = 36.0	<0.001
	Before and After treatment	F(1,86) = 2.2	0.145
	Pop × BfAf	F(2,86) = 4.4	0.015

**N**	5.0 (μM)
Surface fish	18
Pachón cavefish	20
F1 hybrid	30

**N**	5.0 (μM)
Surface fish	35
Pachón cavefish	35
F1 hybrid	19

**Naltrexone-HCl** (10 μg/ body-weight g, repeated two-way ANOVA)			
Sleep duration	Population	F(2,20) = 22.3	<0.001
	Dose	F(1,20) = 5.6	0.028
	Pop × Dose	F(2,20) = 3.7	0.042
Swimming distance	Population	F(2,20) = 18.6	<0.001
	Dose	F(1,20) = 0.8	0.393
	Pop × Dose	F(2,20) = 4.0	0.035
Vibration attraction	Population	F(2,79) = 7.5	<0.001
	Before and After treatment	F(1,79) = 1.0	0.316
	Pop × BfAf	F(2,79) = 2.1	0.125

**N**	10.0 (μg/ body weight g)
Surface fish	8
Pachón cavefish	8
F1 hybrid	7

**N**	10.0 (μg/ body weight g)
Surface fish	31
Pachón cavefish	27
F1 hybrid	24

**Aripiprazole** (1 μM, repeated two-way ANOVA)			
Sleep duration	Population	F(1,45) = 21.1	<0.001
	Before and After treatment	F(1,45) = 3.3	0.074
	Pop × BfAf	F(1,45) = 7.9	0.007
Swimming distance	Population	F(1,45) = 21.5	<0.001
	Before and After treatment	F(1,45) = 69.6	<0.001
	Pop × BfAf	F(1,45) = 39.8	<0.001
Vibration attraction	Population	F(1,44) = 33.6	<0.001
	Before and After treatment	F(1,44) = 7.2	0.010
	Pop × BfAf	F(1,44) = 10.2	0.003

**N**	1.0 (μM)
Surface fish	23
Pachón cavefish	24

**N**	1.0 (μM)
Surface fish	13
Pachón cavefish	33

**Risperidone** (1 μM, repeated two-way ANOVA)			
Sleep duration	Population	F(1,37) = 161.6	<0.001
	Before and After treatment	F(1,37) = 1.4	0.237
	Pop × BfAf	F(1,37) = 3.2	0.08
Swimming distance	Population	F(1,37) = 144.9	<0.001
	Before and After treatment	F(1,37) = 6.5	0.015
	Pop × BfAf	F(1,37) = 4.6	0.038
Vibration attraction	Population	F(1,32) = 34.2	<0.001
	Before and After treatment	F(1,32) = 8.2	0.007
	Pop × BfAf	F(1,32) = 8.4	0.007

**N**	1.0 (μM)
Surface fish	19
Pachón cavefish	20

**N**	1.0 (μM)
Surface fish	15
Pachón cavefish	19

BfAf: Before and after the drug treatment. Pop: population
